# Manipulating molecules with strong coupling: harvesting triplet excitons in organic exciton microcavities†
†The data underlying all figures in this publication are available at https://figshare.com/s/59d2dc8215881c9a157d
‡
‡Electronic supplementary information (ESI) available: Materials and experimental methods, reference sample characterisation, detailed kinetic model. See DOI: 10.1039/c9sc04950a


**DOI:** 10.1039/c9sc04950a

**Published:** 2019-11-27

**Authors:** Daniel Polak, Rahul Jayaprakash, Thomas P. Lyons, Luis Á. Martínez-Martínez, Anastasia Leventis, Kealan J. Fallon, Harriet Coulthard, David G. Bossanyi, Kyriacos Georgiou, Anthony J. Petty, II, John Anthony, Hugo Bronstein, Joel Yuen-Zhou, Alexander I. Tartakovskii, Jenny Clark, Andrew J. Musser

**Affiliations:** a Department of Physics and Astronomy , University of Sheffield , Hicks Building, Hounsfield Road , Sheffield S3 7RH , UK . Email: jenny.clark@sheffield.ac.uk; b Department of Chemistry and Biochemistry , University of California San Diego , La Jolla , California 92093 , USA; c Department of Chemistry , University of Cambridge , Lensfield Road , Cambridge CB2 1EW , UK; d Department of Chemistry , University of Kentucky , Lexington , Kentucky 40506-0055 , USA; e Department of Chemistry and Biochemistry , Cornell University , Ithaca , New York 14853 , USA . Email: ajm557@cornell.edu

## Abstract

Exciton-polaritons are quasiparticles with mixed photon and exciton character that demonstrate rich quantum phenomena, novel optoelectronic devices and the potential to modify chemical properties of materials. Organic materials are of current interest as active materials for their ability to sustain exciton-polaritons even at room temperature. However, within organic optoelectronic devices, it is often the ‘dark’ spin-1 triplet excitons that dominate operation. These triplets have been largely ignored in treatments of polaritons, which instead only consider the role of states that directly and strongly interact with light. Here we demonstrate that these ‘dark’ states can also play a major role in polariton dynamics, observing polariton population transferred directly from the triplet manifold *via* triplet–triplet annihilation. The process leads to polariton emission that is longer-lived (>μs) even than exciton emission in bare films. This enhancement is directly linked to spin-2 triplet-pair states, which are formed in films and microcavities by singlet fission or triplet–triplet annihilation. Such high-spin multiexciton states are generally non-emissive and cannot directly couple to light, yet the formation of polaritons creates for them entirely new radiative decay pathways. This is possible due to weak mixing between singlet and triplet-pair manifolds, which – in the strong coupling regime – enables direct interaction between the bright polariton states and those that are formally non-emissive. Our observations offer the enticing possibility of using polaritons to harvest or manipulate population from states that are formally dark.

## Abstract 2

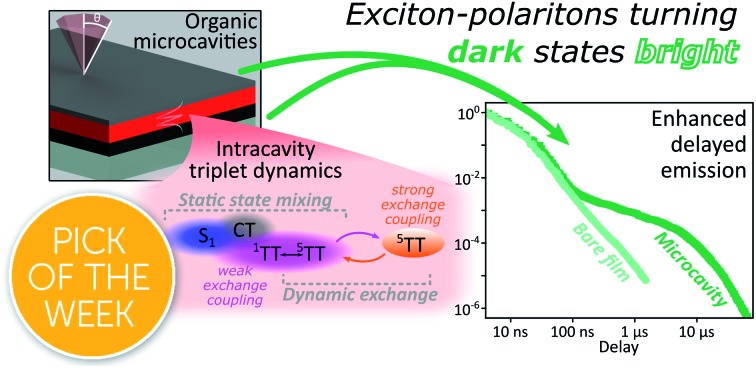
Exciton-polaritons are quasiparticles with mixed photon and exciton character with the potential to modify chemical properties of materials. Here, they are used to provide dark, high-spin triplet-pair states a new pathway to emit light.

## Introduction

**Fig. 1:**
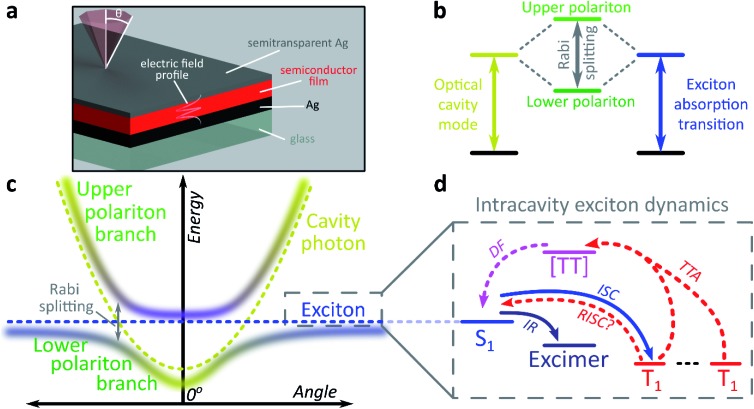
Strong light-matter coupling in optical microcavities. (a) Microcavity structure. A thin film of organic semiconductor or dye dispersed in neutral polymer matrix is deposited in the cavity defined by the two mirrors, here Ag. The thickness of the cavity determines the energy of the confined photonic mode and thus the profile of the electric field inside the cavity, shown here for the λ-mode. Reflection and emission from the cavity are measured as a function of angle *θ*, with 0° defined as normal to the cavity surface. (b) When the cavity mode and the excitonic transition of the semiconductor are near resonance, these two states can couple, forming hybrid upper and lower polariton states. (c) Unlike the exciton (blue), the cavity mode (gold) exhibits distinct angular dispersion. Coupling between the two yields dispersed polariton branches, with characteristic anti-crossing at the exciton energy. Shading indicates the degree of photonic (gold) *vs.* excitonic (blue) character in the state. (d) Typical excitonic processes possible within organic semiconductor films. IR: intermolecular relaxation, (R)ISC: (reverse) intersystem crossing, TTA: triplet–triplet annihilation, DF: delayed fluorescence. Solid arrows indicate processes known to modify exciton-polariton emission dynamics, while dashed arrows show processes not typically explored within microcavities.

The exploration of new material properties typically faces significant practical constraints from cumbersome synthesis and morphological control. In recent years, however, it has been shown that many materials properties can be non-synthetically tuned with confined light fields to form exciton-polaritons^1^–^3^ or vibro-polaritons,^4^–^6^ pointing the way to an entirely new field of microcavity-controlled chemistry.^7^–^11^ These exciton-polaritons are quasi-particles mixing light (photon) and matter (exciton) components, leading to rich quantum effects^12^–^16^ and potential optoelectronic applications.^2^,^3^,^7^,^17^–^21^ Exciton-polaritons are formed by placing a semiconductor or dye between two mirrors to create a Fabry–Perot microcavity in which light of the correct angle and wavelength can be trapped (Fig. 1a). If the material within the cavity has a strong exciton absorption, in resonance with the trapped photon mode, the exciton and photon can couple and form hybrid polariton states (Fig. 1b). As a consequence of the mixed exciton-photonic character of these states, a measurement of reflected light as a function of incident angle demonstrates the typical dispersion shown in Fig. 1c, with the upper polariton branch (UPB) and lower polariton branch (LPB) split around the excitonic energy.

Most studies of exciton-polariton physics have focussed on inorganic semiconductor systems.^12^,^13^,^16^–^18^ In comparison, organic materials have the advantage of high oscillator strength,^22^ affording much stronger light-matter coupling that can result in Rabi splittings between the UPB and LPB in the range 0.1–1e V.^1^,^2^,^22^–^25^ Organics also have low dielectric constants (*ε*_r_ typically 2–4). Consequently, photoexcitation results in bound electron–hole pairs known as Frenkel excitons, with binding energies on the order of 0.5–1 eV. Such high binding energies mean exciton-polaritons are stable and can even condense at room temperature; the latter phenomenon has been observed now in several organic semiconductor microcavities.^14^,^26^,^27^ The tightly bound Frenkel excitons also exhibit complex photophysics, with numerous radiative and non-radiative decay pathways possible following initial photoexcitation (Fig. 1d).^28^ These pathways are also active within microcavities, specifically in the ‘exciton reservoir’ of uncoupled intracavity states, and it is typically expected that they proceed similarly to in free films. They are rarely treated in detail in organic exciton-polariton studies, where the focus is primarily on ‘bright’ singlet (spin-0) excitons. However, it has been shown that the polariton emission dispersion and dynamics can be significantly influenced by population transfer from the reservoir – the parent bright state^29^–^31^ or weakly emissive excimers formed by intermolecular relaxation^32^,^33^ – and theoretical attention increasingly has started to turn to the impact of other non-radiative photophysical processes.^9^–^11^,^34^

We focus here on the role in these systems of triplet (spin-1) excitons. An additional consequence of the low dielectric constant of organic materials is a large exchange energy, which results in the lowest triplet exciton being located >0.5 eV below the first singlet exciton.^35^ Triplet excitons and their management are critical in organic semiconductor devices such as displays and solar cells.^35^–^38^ For example, 75% of excitons formed by electron–hole recombination in optoelectronic devices are triplets due to spin statistics. Triplets are a main reason for the absence of continuous optically pumped organic lasers, but could be useful in solar cells.^37^,^38^ Triplets can be generated from singlet excitons *via* intersystem crossing, which is generally slow (ns or longer) due to weak spin–orbit coupling. Once formed, return to the ground-state requires a spin-flip. Therefore, triplets cannot be directly photoexcited and, in typical organic materials, are non-emissive and long-lived (≫μs).

Only states with large oscillator strength couple to the photon in a microcavity, with triplet states considered a loss channel in organic exciton-polariton systems.^25^ However, states within the reservoir – including triplets, as we demonstrate here – can interact with the polaritons. It has recently been shown that polaritons can be used to alter the decay lifetime of weakly emissive triplet states, and it was suggested this was due to efficient reverse intersystem crossing from the triplets into the lower polariton.^39^ A caveat in that study is that transfer into the lower polariton was not directly resolved, and subsequent theoretical and experimental work indicates that, for a broad range of materials, this process could be less efficient than the intrinsic reverse intersystem crossing channel.^40^,^41^ Moreover, both experimental studies have focused on materials where the triplet state is already partially emissive.^39^,^40^ Similarly, a recent report on tetracene single crystals studied delayed emission caused by triplet–triplet annihilation.^42^ This process is well known in the bulk material and results in substantial emission from the reservoir of triplet states.^43^,^44^ Upon strong coupling to localised surface plasmon resonances the crystal exhibited an enhancement of both prompt and delayed emission in the strong-coupling regime.^42^ The mechanism of this effect is unclear, though the facile conversion between singlet and triplet manifolds suggests that radiative pumping^32^ may play a role. Significantly more work is needed to understand if and how ‘dark’ triplet states – the most common type in organic semiconductors – can interact with the polariton. This problem takes on particular importance in light of the continued progress in electrically injected polariton devices,^18^–^20^ where triplets can no longer be ignored. Just like with electrical injection, a very large reservoir of triplets can be generated by photoexcitation in some materials, making them excellent model systems for the detailed study of polariton–triplet interactions. Large triplet populations can be optically generated in materials with strong spin–orbit coupling resulting in fast intersystem crossing^25^ or in materials in which the exchange energy is so large that the singlet energy is approximately twice the triplet energy. In the latter, photoexcitation into the singlet state results in formation of two triplets through singlet exciton fission.^28^,^45^

We use both intersystem crossing and singlet fission to optically generate triplets and show how triplet excitons interact with polariton states. We find that strong coupling creates entirely new radiative channels that are unavailable in the bare film. The microcavity allows us to extract light from these ‘dark’ triplet states, ‘tilting’ the system towards photon emission in an excitonic analogue to the tuning of chemical reactivity enabled by strong-coupling to molecular vibrational transitions.^6^ This results in ultra-long-lived polariton emission and the potential for harvesting ‘dark’ triplets in devices. We propose a mechanism based on population transfer between the different state manifolds and the new pathways that emerge under strong coupling, and we show that even though the dynamics are dominated by transfer between the dark triplet and singlet states at early times, an efficient channel of conversion of triplet states to light-emitting polariton modes is predominant at long times. The small but non-zero coupling between these dark excitonic states and the polariton stems from the widespread phenomenon of excited-state mixing, which could open the way to using strong light-matter coupling to manipulate the dynamics of states that do not interact directly with light.

## Results

### Polariton emission from triplet–triplet annihilation

**Fig. 2:**
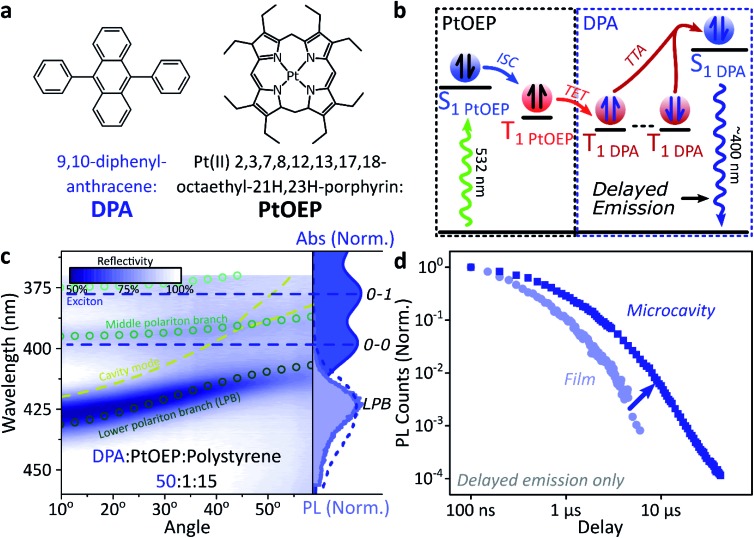
Sensitised photon up-conversion. (a) Molecular structures of active components used in photon up-conversion system. (b) Simplified schematic of photon up-conversion, details in main text. ISC: intersystem crossing, TET: triplet energy transfer, TTA: triplet–triplet annihilation. (c) Reflectivity map of photon up-conversion blend within a Ag–Ag microcavity. Comparison with absorption spectrum (upper right, dark blue) and transfer matrix modelling (lines, circles) confirms strong coupling, characterised by anti-crossings at the 0–0 and 0–1 energies (dashed). Details of transfer matrix model in ESI,‡ Methods. All emission comes from the lower polariton branch (LPB), whether excitation is resonant with diphenylanthracene (355 nm, lower right, dashed spectrum) or PtOEP (532 nm, lower right, shaded spectrum). Emission is collected with a NA = 0.76 lens and thus effectively integrates along the entire dispersion (±45°). (d) Decay kinetics of diphenylanthracene/exciton-polariton emission following excitation of PtOEP at 532 nm reveal enhanced lifetime in microcavity (dark) *vs.* bare film (light). All emission on these timescales arises from triplet–triplet annihilation. Incident power (film: 50 μW, microcavity: 150 μW) yields ∼2*x* greater excitation density in the microcavity, which should result in faster intrinsic depletion of triplets.

A common way to study triplet excitons is through delayed fluorescence, which can occur through the spin-allowed conversion of two triplets into one singlet exciton, known as ‘triplet–triplet annihilation’.^28^,^46^ The resulting emission approximately tracks the ∼μs lifetime of the triplet population rather than the (‘prompt’) ∼ns singlet lifetime. One of the best-characterised triplet–triplet annihilation systems is the blend of diphenylanthracene and metal porphyrins used for photon up-conversion,^47^,^48^ as shown in Fig. 2. We depict the photophysics of this system schematically in Fig. 2b: directly exciting the Pt–porphyrin at 532 nm initiates efficient intersystem crossing (<100 fs),^47^ producing triplets that can transfer to diphenylanthracene where triplet–triplet annihilation produces ‘up-converted’ delayed fluorescence at a significantly shorter wavelength than the original excitation. In solution, the long triplet lifetimes allow this process to occur with high quantum efficiency.^46^,^49^

In order to understand how triplets behave in microcavities, we need to study delayed fluorescence in the solid state, rather than solution. We therefore prepared films of diphenylanthracene/Pt–porphyrin/polystyrene blends with a ratio of 50 : 1 : 15. The polystyrene is used to aid mixing between the two active materials and reduce film roughness. Films and microcavities were encapsulated in inert atmosphere to protect against oxygen quenching of triplets^50^ (see ESI,‡ Methods). The absorption of a control, non-cavity film is shown in Fig. 2c. Emission behaviour of the films is consistent with literature.^47^,^48^ As expected, excitation within the Pt–porphyrin band (532 nm) produces up-converted diphenylanthracene fluorescence (400–500 nm). However, as with other solid-state up-conversion systems, we also observe strong Pt–porphyrin phosphorescence (650 nm) due to phase separation.^47^,^48^

Fig. 2c shows a reflectivity map of a microcavity containing the diphenylanthracene blend, as a function of incident angle and wavelength. The dips in microcavity reflectivity never cross the bare exciton energy (blue dashed). This ‘anti-crossing’ is a signature of strong light-matter coupling and polariton formation, and the absorbing states are thus split into polariton branches. Transfer-matrix modelling based on measured optical parameters confirms strong coupling in this structure to the diphenylanthracene S_1_ state (Fig. 2c, lines and circles). There is no evidence of strong coupling to the much weaker Pt–porphyrin absorption (ESI, Fig S1‡). Microcavity emission originates from the lower polariton branch (Fig. 2c, right), whether we excite the strong-coupled diphenylanthracene directly (355 nm, dashed) or the uncoupled Pt–porphyrin (532 nm, shaded). In the latter case photoexcitation generates a reservoir of uncoupled triplet excitons, therefore the microcavity emission must be due to up-conversion through triplet–triplet annihilation, similar to the single-crystal tetracene result^42^ but without direct photoexcitation of the material which undergoes strong coupling (here, the DPA dark reservoir of S_1_ states).

### Long-lived triplet-derived emission in microcavities

We explore how this triplet harvesting process is affected by strong coupling using time-resolved measurements. Because the processes of interest are intrinsically slow, we apply high-sensitivity gated photoluminescence measurements to resolve the dynamics on long timescales. We observe no change in emission lineshape over the full lifetime (ESI, Fig S11‡). After accounting for the differences in absorbed laser power between film and microcavity samples (details in ESI,‡ Section 1.4), we find that the lifetime of emission in the microcavity is distinctly longer than in the film (Fig. 2d). This is the case even though the microcavity is excited at ∼2*x* higher absorbed laser power, resulting in a 2*x* higher triplet population that, naively, should accordingly undergo faster bimolecular annihilation and exhibit a faster decay of delayed emission. As noted above, all emission on these timescales originates from triplet–triplet annihilation within the reservoir of uncoupled excitons, and we conclude that the microcavity enables harvesting of an additional long-lived species. This change in lifetime is surprising and requires further investigation. We noticed that this ternary blend undergoes laser-induced phase segregation, making detailed studies on this system difficult. We therefore apply the same approach to two simpler reference systems with a single active component.

**Fig. 3:**
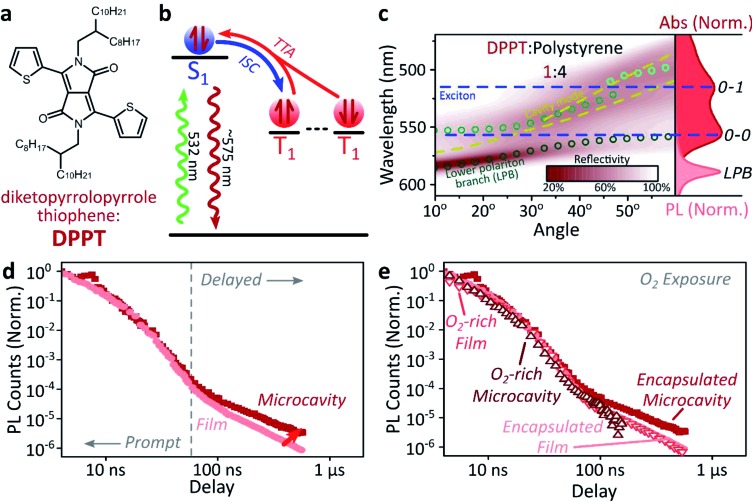
Delayed emission in single-component film. (a) Molecular structure of DPPT. (b) Simplified schematic of DPPT film photophysics, details in main text. ISC: intersystem crossing, TTA: triplet–triplet annihilation. (c) Reflectivity map of DPPT : polystyrene film within a Ag–Ag microcavity. Comparison with absorption spectrum (right) and transfer matrix modelling (lines, circles) confirms strong coupling to the 0–0 transition. All emission arises from the lower polariton branch (LPB). Emission is collected with a NA = 0.76 lens and thus effectively integrates along the entire dispersion (±45°). (d) Integrated photoluminescence kinetics over full emission band for bare film (light) and microcavity (dark) following excitation at 532 nm. Enhanced microcavity emission matches the ‘delayed’ regime, in which contributions from triplet–triplet annihilation are significant. (e) Comparison of encapsulated (solid) and oxygen-exposed (open) films and microcavities reveals nearly identical dynamics in the prompt decay regime. The polariton-induced enhancement on long timescales disappears in the O_2_-exposed microcavity, confirming it arises from triplet excitons. Encapsulated microcavity kinetic is reproduced from panel d.

Diketopyrrolopyrrole thiophene (DPPT, Fig. 3a) is the base unit for polymers exhibiting high charge-carrier mobility in thin-film transistors, recently used in electrical-injection polariton OLEDs.^20^ DPPT monomers are also known to undergo intersystem crossing in the solid state (Fig. 3b).^51^ Films were prepared containing DPPT dispersed in polystyrene matrix (1 : 4 DPPT : polystyrene). Reference photoluminescence measurements on these films reveal delayed fluorescence which is quenched by oxygen and a non-linear intensity dependence, suggesting the weak delayed fluorescence in DPPT results from bimolecular triplet–triplet annihilation. All subsequent measurements were performed on films or microcavities encapsulated in an oxygen-free environment, unless specifically stated.

Within DPPT-based microcavities, Fig. 3c, we observe a clear anti-crossing at the 0–0 peak, while the second peak in the absorption appears to be in the weak/intermediate-coupling regime. Similar to the diphenylanthracene cavities, we attribute the anti-crossing states to polariton branches, and emission is again entirely from the lower polariton branch. Comparison of the film and microcavity emission kinetics in Fig. 3d reveals that the prompt fluorescence dynamics remain unchanged. However, delayed fluorescence from triplet–triplet annihilation once again exhibits a longer lifetime in the microcavity. By contrast, in a reference material INDB in which we observe no contribution to emission in the bare film from triplet–triplet annihilation, we also observe no enhancement of long-lived emission in microcavities (ESI, Fig. S3 and S4‡). Likewise, when we quench the triplets in DPPT through exposure to oxygen,^50^ the polariton-induced enhancement observed in Fig. 3d disappears (Fig. 3e; encapsulated microcavity data is reproduced from panel d).

In short, we observe that the lifetime of delayed emission can be increased through strong light-matter coupling to form exciton-polaritons. This process is directly correlated with the triplet population, and if these dark, uncoupled triplet states are quenched or absent there is no enhancement. We attribute this behaviour to population of polariton states *via* triplet–triplet annihilation from a reservoir of dark, uncoupled triplet excitons, but there must be an additional factor to provide a longer lifetime than observed in thin films. We propose that this effect arises from a distinct longer-lived, non-emissive state that can transfer population to the polariton, namely the high-spin triplet-pair states that arise from the encounter of two spin-1 triplets (see Discussion below). Detailed analysis of the enhancement in diphenylanthracene and DPPT microcavities is complicated by the bimolecular nature of triplet–triplet annihilation: it also depends on (non-uniform) morphology, exciton diffusion and excitation density. We thus turn instead to a system capable of the reverse process, singlet fission, in which a single photon can generate both of the necessary triplets, which then undergo *geminate* triplet–triplet annihilation.^28^,^45^,^52^–^54^

### Singlet fission and quintet harvesting in microcavities

**Fig. 4:**
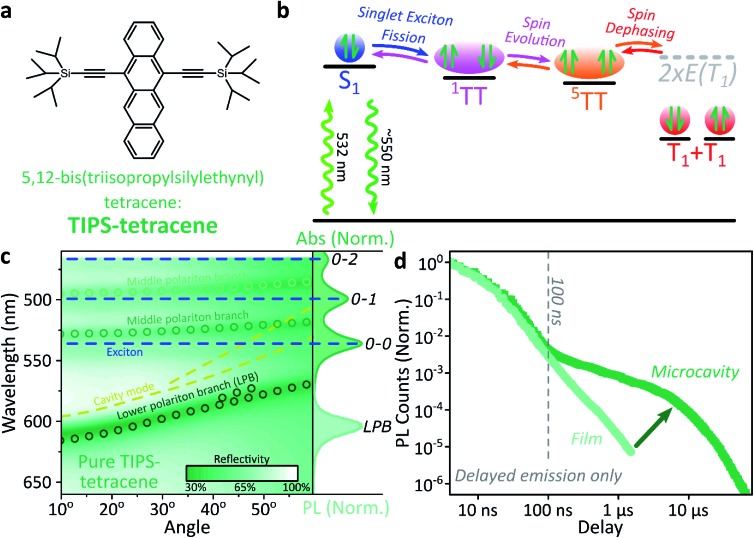
Singlet fission into bound TT within a microcavity. (a) Molecular structure of TIPS-tetracene. (b) Simplified schematic of TIPS-tetracene photophysics, details in main text. All processes are potentially reversible, leading to delayed fluorescence from triplet–triplet annihilation. (c) Reflectivity map of a pure TIPS-tetracene film within Ag–Ag microcavity. Comparison with absorption spectrum (right) and transfer matrix modelling (lines, circles) confirms strong coupling to multiple vibronic transitions. Emission is from the lower polariton branch (LPB). Emission is collected with a NA = 0.76 lens and thus effectively integrates along the entire dispersion (±45°). (d) Integrated photoluminescence kinetics over full emission band for bare film (light) and microcavity (dark) following excitation at 532 nm. All emission on these timescales arises from triplet–triplet annihilation.

We use polycrystalline films of TIPS-tetracene (Fig. 4a), which has been thoroughly characterised with a range of complementary spectroscopic techniques (optically detected magnetic resonance, transient and/or magnetic field-dependent -absorption, -photoluminescence and -EPR).^54^–^57^ These measurements have established a detailed picture of the excited-state and spin-dependent dynamics, which we directly rely on here. Rapid singlet fission from S_1_ generates the spin-0 triplet-pair state ^1^(TT). This can then evolve into higher-spin pair states such as ^5^(TT),^56^,^58^–^60^ as well as a mixed-spin state ^1^(TT)–^5^(TT),^55^ on sub-μs timescales. Unlike the bimolecular triplet–triplet annihilation above, this evolution does not depend on exciton diffusion, allowing us to temporally isolate the contribution of high-spin states in microcavities.

**Fig. 5:**
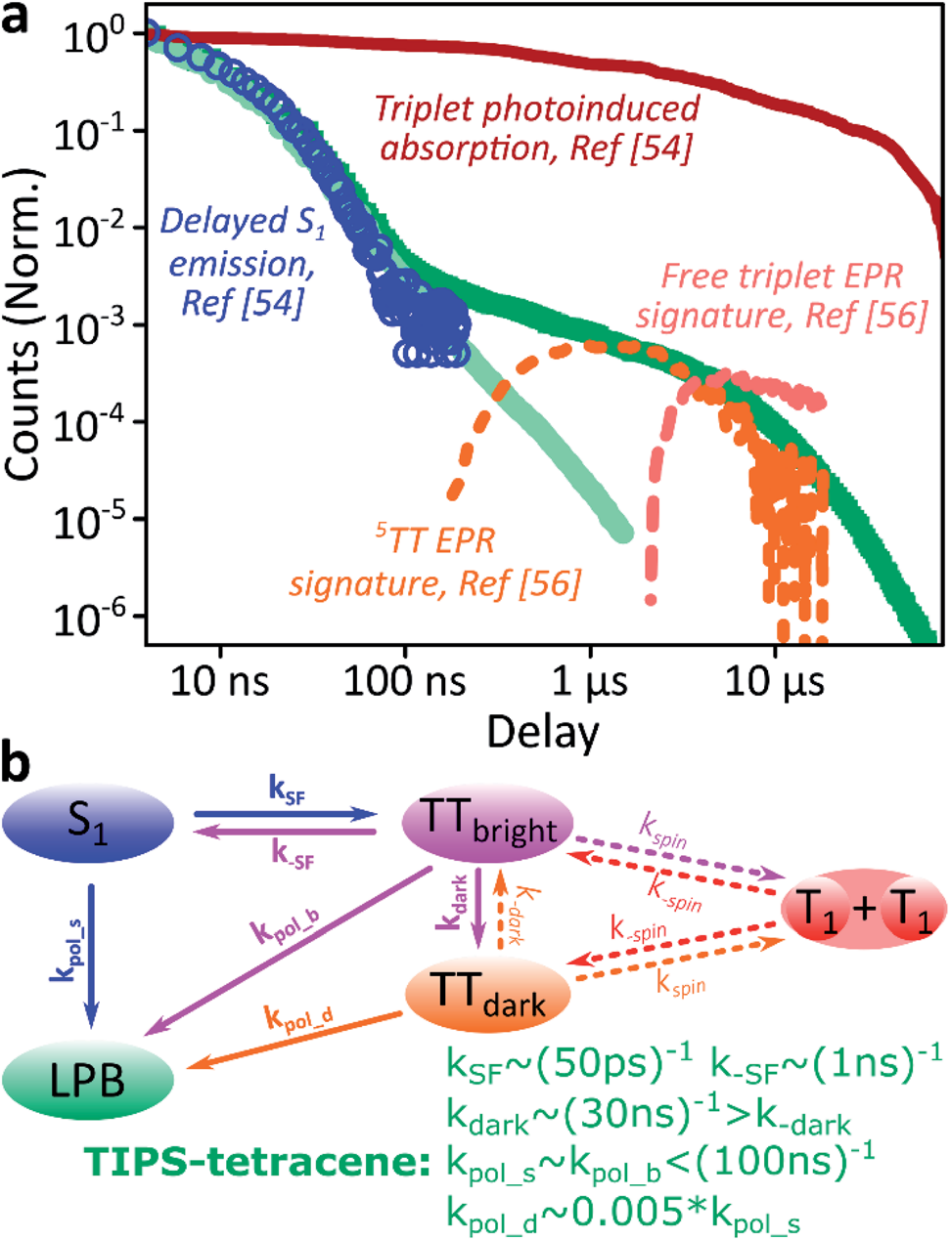
Identification of the ^5^TT contribution. (a) Reproduction of the TIPS-tetracene film and microcavity emission kinetics from Fig. 4d, compared with time-correlated single photon counting (circles, ref. 54), transient absorption (solid, ref. 54) and electron paramagnetic resonance (dashed, ref. 56) kinetics previously reported for equivalent polycrystalline films. The long-time enhancement in microcavities coincides with observations of high-spin ^5^(TT). (b) Rate model used to describe dynamics within the exciton reservoir. TT_bright_ are triplet-pair states that directly mix with S_1_. TT_dark_ states are unable to efficiently repopulate S_1_. Rates shown are from fitting to published data. A similar model would describe diphenylanthracene : PtOEP or DPPT, replacing *k*_SF_ with *k*_ISC_ directly to the (single) triplet states and including the effects of exciton diffusion within *k*_spin_. Nonradiative decay (from all states) and radiative decay (from S_1_, LPB) omitted for clarity.

Just as in the photon up-conversion blend and DPPT, we consider the photophysical dynamics of the thin film to be active also within the exciton reservoir of our microcavities, and we outline the basic progression in TIPS-tetracene here (Fig. 4b). In these films, singlet fission occurs within 50 ps,^54^ well within our instrument response of 4 ns. The singlet and ^1^(TT) states are very similar in energy resulting in a dynamic equilibrium and weak delayed fluorescence. Over time, this bound triplet-pair state adopts increasing ^5^(TT) character^56^ and the films become non-emissive. This explains the fact that while the total triplet-pair population, probed with transient absorption spectroscopy (Fig. 5, solid line), does not decay significantly over μs timescales, the delayed fluorescence (Fig. 5, open circles) does.^54^ It also explains the presence of ^5^(TT) on microsecond timescales, probed with time-resolved electron paramagnetic resonance (Fig. 5, dashed line).^56^ This process probably relies on off-diagonal dynamic disorder or triplet-hopping that promotes fluctuations in the spin-exchange coupling,^61^–^64^ see discussion below. On very long timescales (>μs), spin dephasing yields pairs of triplets with independent spins that nonetheless remain bound.^54^,^56^ Due to this binding, the predominant triplet–triplet annihilation processes in TIPS-tetracene are geminate on all timescales,^54^ meaning annihilation occurs between triplets formed from the same parent singlet state and the same absorbed photon. As a result, the normalised delayed fluorescence kinetics show no dependence on excitation density allowing straightforward direct comparison of microcavity and film dynamics.

Within microcavities we observe strong coupling throughout the TIPS-tetracene absorption band and clear polariton branches, Fig. 4c. As above, emission is predominantly from the bottom of the lower polariton branch independent of the energy offset between S_1_ and the cavity photon at 0°, the ‘detuning’. Relative to the bare film, the pre-100 ns microcavity emission is only weakly perturbed, Fig. 4d. However, *beyond 100 ns the microcavity shows a significantly enhanced long-lived tail*.

This effect is qualitatively reproduced for a wide range of exciton-photon detunings, though the magnitude of enhancement varies between microcavities (ESI, Fig. S9‡), and the polariton population distribution along the LPB points to the existence of multiple broad energetic resonances (ESI, Fig. S10‡). We observe the same behaviour in samples where the TIPS-tetracene active layer is physically separated from the Ag mirrors with 20 nm spacer layers (ESI,‡ Section 4.2). This effect cannot be induced through any of the individual sample fabrication steps (ESI,‡ Section 4.1) but only occurs in full strongly coupled structures. As in previous systems, the film and microcavity spectral shapes exhibit negligible evolution over this decay (ESI, Fig. S11‡). In the film this is because all emission we detect is from S_1_, populated by triplet–triplet annihilation from ^1^(TT).

Likewise, in the microcavity the constant spectral shape indicates that the emission is mediated by the lower polariton state, also populated by annihilation of triplet pairs in the reservoir. However, the clear delineation into two kinetic regimes in Fig. 4d suggests that on long times (>100 ns) this process follows a distinct pathway that is unavailable in the film. To identify this pathway, in Fig. 5a we compare our results with published data from TIPS-tetracene polycrystalline films that show identical absorption spectra and fluorescence lifetimes to our own and are thus equivalent.^54^,^56^ Interestingly, the bulk of our microcavity-enhanced emission coincides with the time frame when quintet states are observed in the film (dashed line), and we conclude that the lower polariton can be directly populated by the quintet pairs.

We can describe the effect of strong coupling with the simple kinetic model illustrated in Fig. 5b (see ESI,‡ Section 5, for details). Here, TT_bright_ denotes well-defined triplet-pair states able to couple directly to S_1_ (*e.g.*^1^(TT)); these states quickly establish a thermal equilibrium with S_1_ upon which spin evolution of the pair occurs.^54^ TT_dark_ includes all pair states that do not directly or strongly interact with S_1_, such as the previously observed ^5^(TT) state and the mixed-spin ^1^(TT)–^5^(TT) state. Using published data^54^,^56^,^65^ to fix the forward and reverse singlet fission rates *k*_(–)SF_, the conversion rates between bright and dark TT states *k*_(–)dark_ and the rate of spin dephasing into the uncoupled pair (T_1_ + T_1_) *k*_(–)spin_, we arrive at a highly constrained model that can simultaneously describe all reported neat-film TIPS-tetracene dynamics,^54^–^56^ including our own data. To describe the situation in our microcavities, we include the new emissive LPB state and use the same intrinsic rate constants for the photophysics involving states within the exciton reservoir. The only new rate constants that appear, *k*_pol_x_, describe the transfer of population from the exciton reservoir states x to the LPB. We have identified only two scenarios that agree with our experimental results for the microcavity. (1) The rate constant *k*_–dark_ is greatly enhanced, enabling harvesting of the large TT_dark_ population *via* S_1_, while *k*_pol_d_ is negligible. The LPB in this regime is only directly populated from relaxation of singlet states in the reservoir. This would mean that strong coupling to S_1_ perturbs the spin physics of uncoupled states within the cavity. Alternatively, (2) *k*_pol_d_ > 0 and all other rates remain the same, allowing direct transfer from the reservoir of TT_dark_ excitons into the LPB. While both routes are very surprising, we consider the *k*_pol_d_ pathway to be much more probable, and it provides a better fit to the full dataset (ESI, Fig. S15‡). The chief effect of strong coupling is thus to open an energetically downhill pathway for population transfer from TT_dark_ into the emissive LPB. Let us briefly abound in this latter proposed scenario.

It is interesting that given the long lifetime of the TT_dark_ excitons and the fast cavity leakage rate, the TT_dark_ → LPB rate does not need to be large to enable a large effect in the delayed photoluminescence. The associated downhill population transfer can occur *via* a combination of radiative pumping or *via* nonadiabatic transfer mechanisms, where the rate-limiting step is respectively mediated by photonic or matter components. In radiative pumping, a small S_1_ fraction of the TT_dark_ manifold allows for the emission of phonons and a red-shifted virtual photon^52^ that is absorbed by the LPB; in nonadiabatic transfer, the electronic coupling between TT_dark_ and LPB mediates the transfer of population accompanied by emission of phonons.^41^ In either mechanism, there needs to be a finite electronic coupling between TT_dark_ to S_1_ which, although expected to be very small, is large enough to provide the physical mechanism whereby the TT_dark_ reservoir can directly feed the LPB.

## Discussion

In all the systems we have studied in which triplets are formed, triplet–triplet annihilation leads to longer-lived emission in the strong-coupled microcavity compared with the bare film. Because of the long timescales involved, this is a significant effect even though the prompt emission from these states is weak. Integration of the kinetics in Fig. 2–4 suggests a polariton-induced enhancement to the total emission of up to 150% (ESI, Fig. S16‡). Based on the model system TIPS-tetracene, and the fact that in all systems in which we observe microcavity-enhanced emission on these timescales the primary species are uncoupled triplet excitons, we have suggested that the enhanced emission comes from harvesting ^5^(TT) into the lower polariton. This result is entirely unprecedented: direct interaction between these pair-states and the polariton is spin- and symmetry-forbidden, and thus very weak. Thus, our observations show that strong coupling can alter the photophysics even of states that cannot interact directly with light.

### Theoretical foundations of the kinetic model

**Fig. 6:**
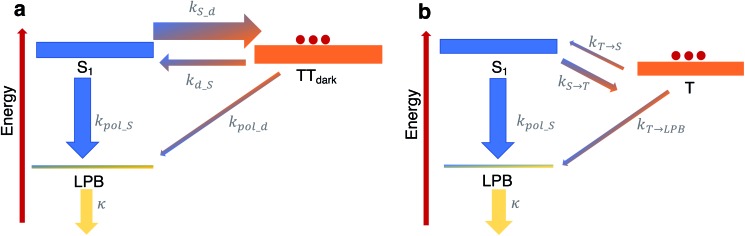
Relevant relaxation pathways within the microcavity. The significant difference between the density of states of the (dark) exciton reservoirs (thick rectangles) and the polariton branches (thin rectangles) renders the transitions between the exciton reservoirs (thick arrows) faster than transfer of population from a reservoir to polariton modes (thin arrows). (a) In TIPS-tetracene, we argue that a large rate constant *k*_S_d_ relative to the corresponding one for the inverse process *k*_d_S_ ≪ *k*_S_d_ effectively traps the equilibrated population in the TT_dark_ manifold. The most efficient channel of photon emission would be through the direct transfer to the LPB, in spite of *k*_d_S_ ≫ *k*_pol_d_, since the leakage rate *k* ≫ *k*_pol_d_ releases photons out of the cavity very quickly. For simplicity, our scheme here omits the TT_bright_ manifold (see Fig. 5b) since we consider the long-time limit where the delayed photoluminescence is determined by the population in TT_dark_. (b) In contrast to our results, in the scenario of a faster feeding of the LPB by the dark S_1_ manifold compared to the timescales of transition between triplet T and dark S_1_ states (as in thermally-activated delayed fluorescence chromophores), the delayed photoluminescence signal is, in general, expected to be the same in both microcavity and cavity-free scenarios, since its decay profile is determined by the rate-limiting step associated with *k*_T→S_.

To shed more insight into the experimental results above, we highlight the essential features of the kinetic model (see Fig. 6a, which is a simplified version of Fig. 5b). As emphasized before, we consider rate constants of transfer between dark exciton manifolds as identical to the corresponding rate constants in the cavity-free case. We also expect the rates that account for transfer from dark exciton reservoirs to polariton modes (*k*_pol_x_) to be smaller than their corresponding out-of-cavity analogues (*e.g.* population transfer between TT_dark_ and S_1_) by *N*_eff_ ≈ O(10^6^)–O(10^9^) to account for the deleterious effect of delocalization of the final polariton states.^40^ This dilution factor can be intuitively explained through the mismatch in wavefunction delocalization of these states: in the presence of disorder, the dark excitons can be regarded as essentially localized on particular molecular sites while polaritons are delocalized through the coherence length of the optical cavity mode, so the overlap between initial and final states scales as 1/*N*_eff_. These ‘direct’ processes between dark states and the polariton modes may thus seem strongly disfavoured but, as we shall argue, they play a crucial role in the long-time photophysics of the presently studied organic polariton systems.

Another important fact to consider is that singlet fission in TIPS-tetracene occurs on the picosecond timescale,^54^ whereas the triplet–triplet annihilation process lifetime is on the order of nanoseconds,^65^ the latter being approximately one order of magnitude faster than the fluorescence lifetime of the bright singlet excitons (see Fig. 6a and ESI‡ for the rate constants used in our model and their justification). In the bare film scenario, these conditions imply that a thermal equilibrium which is heavily biased towards the exciton reservoir of TT_bright_ states is quickly established well before emission of a photon occurs *via* the S_1_ states. The additional irreversible transfer from TT_bright_ to TT_dark_ states leads to the accumulation of population in the latter. Once most of the initial population is trapped in the TT_dark_ manifold, the fluorescence signal is negligible as a result of the slow back transfer from TT_dark_ to TT_bright_ states, and the presumably small (effective) coupling between TT_dark_ and S_1_ states. This behavior is captured in Fig. 6a by the coarse-grained effective rate constants *k*_S_d_ and *k*_d_S_, which are heavily biased towards TT_dark_ (these effective rate constants are implicit functions of *k*_SF_, *k*_–SF_, *k*_dark_, *k*_–dark_ and therefore, should be understood as incorporating the mediating role of TT_bright_, which we omit for simplicity).

While the coupling between TT_dark_ and S_1_ states is small and irrelevant in the cavity-free case, it turns out to be of prime relevance inside of the microcavity as it provides the only direct channel to funnel TT_dark_ population into the LPB. This process is guaranteed to be slow due to the small electronic mixing between TT_dark_ and S_1_, as well as the large dilution factor of *N*_eff_. Yet, it is followed by a very fast photonic leakage *via* the photonic component of LPB. Thus, photon emission *via* the direct pathway TT_dark_ → LPB ends up being more efficient than its TT_dark_ → S_1_ → LPB counterpart, where the equilibrium of dark S_1_ ↔ TT_dark_ (largely biased towards TT_dark_) precludes the population trapped in TT_dark_ to reach the dark S_1_ states in due time (see Fig. 6a). This biased equilibrium implies that this detrapping of TT_dark_ population *via* the LPB is expected to occur even when TT_dark_ → S_1_ (which is already small) is faster than TT_dark_ → LPB.

Our present result seems at odds with previous reports showing that the LPB does not necessarily offer an advantage on the harvesting of delayed fluorescence from triplets in organic materials, even when the lower polariton branch is below the triplet states.^40^ To understand this apparent discrepancy, we notice the difference in timescales between singlet fission/triplet–triplet annihilation (relevant in the present experiment) and those for reverse- and intersystem-crossing (which are the mechanisms that come into play for individual triplet harvesting; see Fig. 6b). Both reverse- and intersystem crossing processes are on the μs timescale,^66^ three orders of magnitude slower than the fluorescence lifetime of singlets. Upon strong coupling, the population trapped in the dark triplet states gives rise to photoluminescence through the most efficient channel, which in this case corresponds to the T → S_1_ → LPB pathway, where T accounts for the triplet states. The direct T → LPB pathway is unable to compete with the indirect pathway in view of the large density of states of the T manifold compared to the LPB. In other words, the T population is not as efficiently trapped in Fig. 6b as the TT_dark_ population is in Fig. 6a, so the direct T → LPB pathways offers no incentives to be utilized. In consequence, the expected decay profile of the microcavity photoluminescence follows the same timescale as that for the cavity-free case, given that the rate-limiting step in both cavity-free and cavity cases is the very slow reverse intersystem crossing channel from triplet to singlet reservoirs. To summarize, the mechanism in Fig. 6b is in sharp contrast with our results, where the predominant mechanism of photoluminescence is different in the microcavity compared to the bare film case.

### Role of mixed adiabatic states

A critical element in cavity mediated harvesting of TT population in TIPS-tetracene and related systems is that there must be non-zero electrostatic coupling between the high-spin pair TT states and the polariton. The process of singlet fission is generally described in simplistic terms, where a singlet exciton splits into a pair of triplet excitons which are initially coupled into a triplet pair. In reality, these excited states are not pure diabatic states but rather mixed adiabatic states. Thus the nominal S_1_ state is often substantially mixed with higher-lying charge-transfer excitons (CT)^67^ and even attains some TT character. The ^1^(TT) state is also substantially mixed with CT and S_1_, resulting in significant binding with respect to two free triplets^52^,^53^,^68^–^70^ (essential for fission to even be possible in TIPS-tetracene^54^) and providing a channel for this formally dark state to emit directly.^52^–^54^,^70^ The mixed character of both states enables ultrafast singlet fission^52^,^54^,^71^,^72^ and efficient equilibration between (adiabatic) S_1_ and ^1^(TT).^54^ Once formed, the adiabatic ^1^(TT) state can exhibit further evolution. The constituent triplets within the pair can interact through exchange coupling. In the regime of weak exchange coupling, the initially created ^1^(TT) state mixes with other spin configurations *via* dipolar interactions, resulting in a triplet pair with mixed singlet and quintet character.^73^ This regime has been detected in TIPS-tetracene using magnetic resonance techniques,^55^ and it is likely that the formation of this dark ^1^(TT)–^5^(TT) mixed state is responsible for the ∼10 ns decay of delayed fluorescence in films^54^ as the state loses its (pure) singlet character. The recent observation in the same and other materials of strongly exchange-coupled triplet pairs suggests that the exchange interaction must fluctuate over time.^56^,^58^–^60^,^62^,^74^ The coexistence of these two regimes on similar timescales indicates that the weakly and strongly exchange-coupled triplet pairs are in dynamic equilibrium, probably driven by thermal fluctuations in the exchange interaction (off-diagonal dynamic disorder).^61^ Importantly, the physical mechanism behind the fluctuating exchange coupling is not linked to singlet fission but is an intrinsic property of organic materials. We can thus expect comparable spin evolution to occur in triplet–triplet annihilation systems, where the first step is formation of a TT encounter complex.

## Conclusions

**Fig. 7:**
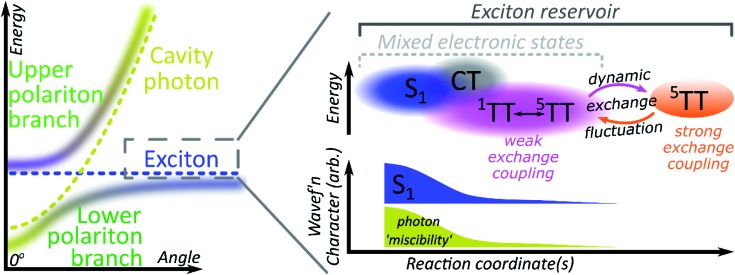
Mixed electronic states within a microcavity. Illustrative schematic of the energy landscape and mixed electronic states that contribute to the reservoir of dark excitons. The latter shall be understood as composed of states with chemical character varying on a continuum along a generalised reaction coordinate, *e.g.* exhibiting increasing mixing with CT and TT states. While the adiabatic states are often named according to their predominant character, other states also contribute to their wavefunctions. Here, mixing with S_1_ allows ‘miscibility’ with the photon through the S_1_-polaritons. The coupling of the pure ^5^(TT) state to the photon is small, yet crucial to the cavity mediated harvesting of TT population demonstrated in this study.

We see, then, that all of the relevant electronic states in the singlet fission pathway are connected by excited-state mixing, as shown in Fig. 7. Mixed adiabatic states are a critical driver in photophysical processes such as ultrafast intersystem crossing,^75^ thermally activated delayed fluorescence,^76^ singlet exciton fission^52^,^54^ and its reverse, triplet–triplet annihilation. This property is a critical distinction from inorganic semiconductors, but it is rarely considered explicitly in the exciton-polariton field. Thus, when the photon couples to S_1_ to form polaritons, it in fact interacts with all the states that mix with S_1_. Consequently, all states that mix with S_1_ may acquire some ability to interact with the polaritonic states. In polaritonic systems in which triplet–triplet annihilation occurs, this creates a pathway to populate the radiative lower polariton branch and thus a route to harvest light from nominally dark diabatic states. Whether the mechanism of polariton feeding proceeds through a radiative (photon-mediated) or a nonradiative (material-mediated) will require further research.

The matrix element coupling the dark pair-states to S_1_ must exist even in the neat film, but the coupling is evidently too small to overcome the energetic or kinetic barriers against delayed emission, and very little is detected. The microcavity offers changes to the energetic structure and reorganisation energies,^40^,^41^ as well as additional radiative and nonradiative rates (Fig. 5 and 6) that enable even this weak coupling element to contribute significantly to emission. While the above explains any microcavity emission originating from ^1^(TT), it does not on its own explain our observation of ^5^(TT) harvesting. Our cavity modified kinetics reveal a correlation with the strongly exchange-coupled, pure ^5^(TT) state. The latter exists in equilibrium with the weakly exchange-coupled ^1^(TT)–^5^(TT) mixed state,^55^,^56^,^61^,^73^ which itself can mix with the photon through its ^1^(TT) component. Hence, in the strong exciton-photon coupling regime, mixed states gain a weak but non-zero coupling element to populate the emissive lower polariton branch. Thanks to dynamic fluctuations in the exchange coupling, this allows even completely dark states like ^5^(TT) to serve as a reservoir for polariton emission.

Interestingly, this behaviour could allow for an improvement in solid-state photon up-conversion. Simple spin considerations suggest that the maximum quantum efficiency of up-conversion should be ∼11%, as only 1 of 9 possible TT pair states/encounter complexes have zero spin (*i.e.*^1^(TT)). Distinctly higher efficiencies have been observed in solution, attributed to the higher spin-states such as ^5^(TT) dissociating without significant loss to reform ^1^(TT).^46^,^77^ This has never been observed in the solid state. In an optimised fluorescence up-conversion system, the ability to directly harvest ^5^(TT) encounter complexes in the weak spin-interaction regime should boost the maximum efficiency. At the same time, the resulting up-converted polariton emission would be well-directed, thereby simplifying collection. A similar mechanism could be used in electrically injected polariton LEDs and lasers, where triplet excitons constitute 75% of the population and triplet–triplet annihilation could be used to harvest them. Because these states are very long-lived, they can make a substantial contribution to the total emission even if the instantaneous probability to emit is always low.^78^ The ability of these very long-lived states to populate the lower polariton can also enable new applications in polaritonic physics. For example, it may be possible to use such a reservoir of non-coupled states to feed a polariton condensate, increasing its effective lifetime. This may be equivalent to the continuous pumping of exciton reservoir states in GaAs microcavities to continually repopulate the polariton condensate.^13^ Such long-lived condensates would be important for practical applications of room-temperature polariton lasing. This concept also vastly expands the scope of microcavity-controlled chemistry, which seeks to alter material properties and light-induced dynamics through strong light-matter coupling.^1^–^3^,^6^–^11^ The interactions implicit in the adiabatic picture mean that strong coupling may perturb not only the state that dominates the absorption spectrum, but also some states that mix with it. Our work presents new opportunities to control processes in which an absorbing state mixes with dark states, for example charge transfer, biological light harvesting, energy transfer and intersystem crossing.

## Author contributions

DWP, HC, TPL and AJM performed the optical experiments. RJ performed transfer-matrix modelling. LAMM and JYZ performed theoretical analysis. AL, KJF, AJP, JA and HB synthesised materials. DWP, DGB and KG prepared samples. DWP, JC and AJM analysed the data and wrote the manuscript. AJM conceived the project and supervised it with JC. All authors discussed the results and commented on the manuscript.

## Conflicts of interest

There are no conflicts to declare.

## Supplementary Material

Infographic

Supplementary information
