# Supplementary material for: Manipulating molecules with strong coupling: harvesting triplet excitons in organic exciton microcavities
Source: Chem Sci. 2019 Nov 27;11(2):343–54. doi: 10.1039/c9sc04950a (PMC7067247; doi:10.1039/c9sc04950a)

Electronic Supplementary Material (ESI) for Chemical Science.
This journal is © The Royal Society of Chemistry 2020

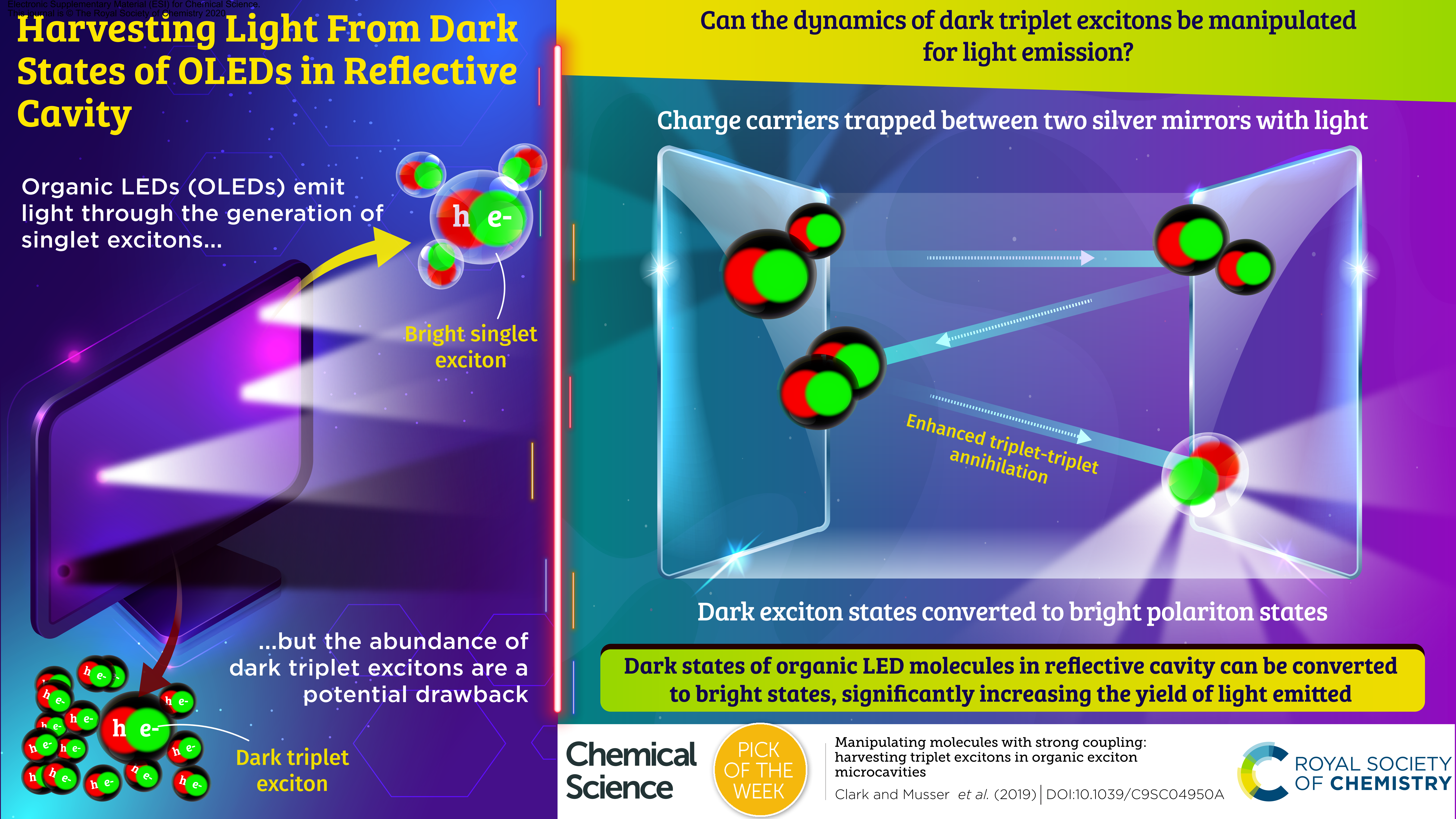

Supplement: Supplementary file 1 [file SC-011-C9SC04950A-s001.pdf]
