# Supplementary material for: Manipulating molecules with strong coupling: harvesting triplet excitons in organic exciton microcavities
Source: Chem Sci. 2019 Nov 27;11(2):343–54. doi: 10.1039/c9sc04950a (PMC7067247; doi:10.1039/c9sc04950a)
Supplement: Supplementary file 2 [file SC-011-C9SC04950A-s002.pdf]

Electronic Supplementary Material (ESI) for Chemical Science.
This journal is © The Royal Society of Chemistry 2020 ESI updated 04/02/2020

Supplementary Information for 'Manipulating molecules with strong coupling: harvesting triplet excitons in organic exciton microcavities'

Daniel Polak[1], Rahul Jayaprakash[1], Thomas P Lyons[1], Luis A. Martinez-Martinez[2], Anastasia Leventis[3], Kealan J Fallon[3], Harriet Coulthard[1], David G Bossanyi[1], Kyriacos Georgiou[1], Anthony J Petty II[4], John Anthony[4], Hugo Bronstein[3], Joel Yuen-Zhou[2], Alexander I Tartakovskii[1], Jenny Clark[1]* and Andrew J Musser[1,5]*

[1] Department of Physics and Astronomy, University of Sheffield, Hicks Building, Hounsfield Road, Sheffield S3 7RH, UK

[2] Department of Chemistry and Biochemistry, University of California San Diego, La Jolla, California 92093, USA

[3] Department of Chemistry, University of Cambridge, Lensfield Road, Cambridge CB2 1EW, UK

[4] Department of Chemistry, University of Kentucky, Lexington, Kentucky 40506-0055, USA

[5] Department of Chemistry and Biochemistry, Cornell University, Ithaca, New York 14853, USA

*email: ajm557@cornell.edu, jenny.clark@sheffield.ac.uk

Contents

1. Methods 3
  1.1. Materials 3
    1.1.1. INDB synthesis 3
    1.1.2. DPPT synthesis 8
    1.1.3. TIPS-tetracene synthesis 9
  1.2. Sample preparation 10
  1.3. Steady-state measurements (absorption, PL, reflectivity) 10
  1.4. Time-resolved photoluminescence 11
  1.5. Transfer matrix simulations 12
2. Diphenylanthracene:Pt-porphyrin:polystyrene 50:1:15 13
3. Indolonaphthyridine benzene (INDB) – Triplet-free reference 15

4. TIPS-tetracene 17
4.1. Solution-processed control samples 18
4.2. Fully evaporated control samples 19
4.3. Detuning dependence 21
4.4. Spectral/k-space evolution 24
5. Rate modeling 25
6. Kinetic enhancement 32
7. References 33

1. Methods

1.1. Materials

Diphenylanthracene, Pt-porphyrin, polystyrene, bathocuproine and all solvents were purchased from Sigma Aldrich and used without further purification. All samples were prepared on ultraflat glass slides coated with 20 nm synthetic quartz (Osilla). INDB, DPPT and TIPS-tetracene were synthesised as follows.

1.1.1. INDB synthesis

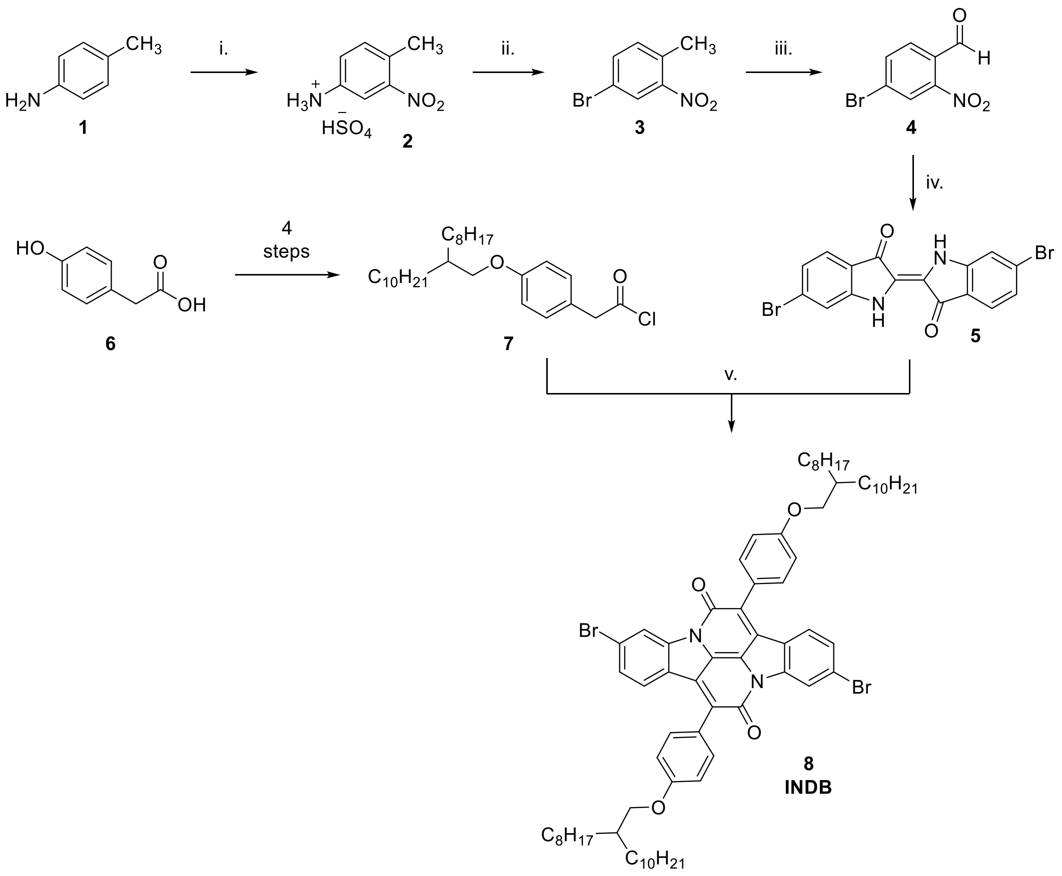

3-Nitro-4-methylphenylammonium hydrosulfate (2)

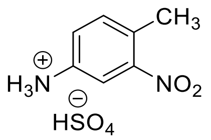

Under normal atmospheric conditions, *p*-toluidine (40.0 g, 0.37 mol) was cautiously dissolved in conc. H_2SO_4 (110 mL) and cooled to –10 °C. A solution of nitric acid (19 mL, 0.43 mol, 70%) in conc. H_2SO_4 (70 mL) was added dropwise insuring the internal temperature did not rise above – 5 °C. After 6 h the reaction was poured onto crushed

ice (1 kg) precipitating a yellow solid which was collected by vacuum filtration and washed with cold water. The solid was dried thoroughly to give a pale-yellow solid (76.9 g, 83%).

A sample of solid was converted to 3-nitro-4-methylaniline for analysis.

^{1}H NMR (600 MHz, $CDCl_3$) δ 7.28 (d, *J* = 2.5 Hz, 1H), 7.07 (d, *J* = 8.2 Hz, 1H), 6.80 (dd, *J* = 8.2, 2.5 Hz, 1H), 3.84 (s, 2H), 2.45 (s, 3H) **LRMS** (CI$^+$) *m/z* 153 $[MH]^+$

4-Bromo-2-nitromethylbenzene (3)

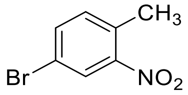

Under normal atmospheric conditions, 3-nitro-4-methylphenylammonium hydrosulfate (29.5 g, 0.12 mol) was dissolved in water (129 mL) and hydrobromic acid (38.5 mL, 0.34 mol, 48%) and cooled to –5 °C. A cold solution of sodium nitrite (15.2 g, 0.22 mol) in water (40 mL) was added slowly, maintaining the temperature below 0 °C, and the reaction stirred for 30 min. Separately, copper(I) bromide (17.2 g, 0.12 mol) in hydrobromic acid (23.8 mL, 0.21 mol, 48%) was heated to reflux. The cold diazonium salt was added portionwise to the copper solution, liberating gaseous nitrogen dioxide. Following complete addition, the reaction was heated at reflux for 2 h, cooled and extracted with diethyl ether (3 × 100 mL). The combined organic extracts were successively washed with 5% ammonium hydroxide solution (2 × 100 mL), water (100 mL) and brine (100 mL), dried over $MgSO_4$ and concentrated *in vacuo* to give a brown oil from which a brown solid separated. Recrystallisation from ethanol yielded 14.8 g (57%) of the title compound as a brown crystalline solid.

^{1}H NMR (600 MHz, $CDCl_3$) δ 8.12 (d, *J* = 2.0 Hz, 1H), 7.62 (dd, *J* = 8.2, 2.0 Hz, 1H), 7.23 (d, *J* = 8.2 Hz, 1H), 2.55 (s, 3H) **LRMS** (CI+) *m/z* 217/215 $[MH]^+$

4-Bromo-2-nitrobenzaldehyde (4)

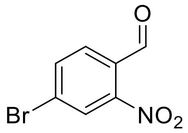

To a solution of 4-bromo-2-nitromethylbenzene (27.5 g, 0.13 mol) in anhydrous DMF (130 mL) under argon was added *N*,*N*-dimethylformamide dimethyl acetal (50.6 mL, 0.38 mol). The reaction was heated at 135 °C for 24 h. Separately, sodium periodate (81.5 g, 0.38 mol) was dissolved in a 2:1 mixture of H_2O:DMF (380 mL). The cooled red enamine intermediate added dropwise to the vigorously stirring periodate solution at RT. If stirring became prevented due to viscosity, H_2O/DMF (2:1) was added until stirring resumed. The reaction was stirred for 3 h at RT and then filtered. The filter cake was washed thoroughly with toluene (300 mL). From the filtrate, the organic layer was separated and washed

with water (2 × 200 mL) then brine (2 × 200 mL), dried over $MgSO_4$ and concentrated *in vacuo* to give an oil to which hexane was added to crystallise the aldehyde (19.6 g, 67%). If the reader obtained a solid following concentration, the crude residue is best purified by dry-flash chromatography eluting with 10% ethyl acetate in hexane.

^{1}H NMR (400 MHz, $CDCl_3$) δ 10.39 (s, 1H), 8.27 (d, *J* = 1.8 Hz, 1H), 7.93 (dd, *J* = 8.3, 1.4 Hz, 1H), 7.85 (d, *J* = 8.3 Hz, 1H) **LRMS** (CI+) *m/z* 232/230 $[M]^+$

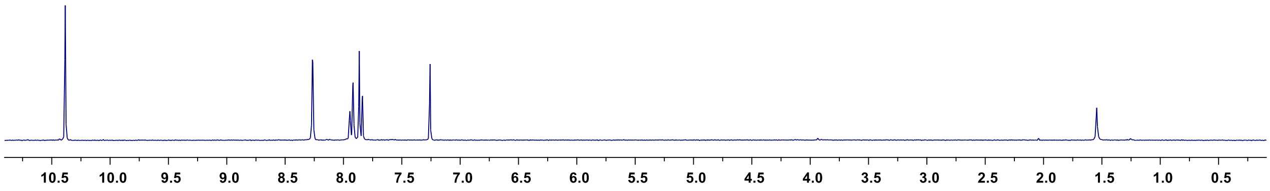

(*E*)-6,6'-dibromo-[2,2'-biindolinylidene]-3,3'-dione (6,6'-dibromoindigo) (5)

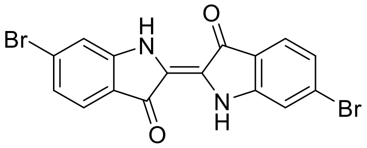

Under normal atmospheric conditions, 4-bromo-2-nitrobenzaldehyde (20 g) was dissolved in acetone (200 mL) and water (200 mL) was added dropwise with vigorous stirring to create a fine suspension. A 1M sodium hydroxide solution (100 mL) was added dropwise, precipitating the purple indigoid. Once addition was complete the reaction was stirred overnight then filtered and washed with acetone. The purple solid was dried thoroughly to give 10.4 g of the title compound (58%).

^{1}H NMR (600 MHz, DMSO) δ 7.57 (d, *J* = 8.1 Hz, 2H), 7.55 (s, *J* = 1.1 Hz, 2H), 7.12 (d, *J* = 8.1 Hz, 2H) **HRMS** Found (EI): $[M]^+$ 417.8957, $C_{16}H_8Br_2N_2O_2$ requires 417.8953

Ethyl 2-(4-hydroxyphenyl)acetate (6)

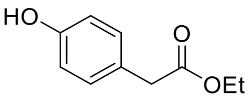

Under normal atmospheric conditions, a solution of 2-(4-hydroxyphenyl)acetic acid (10 g, 65.7 mmol) in ethanol (120 mL) and conc. H_2SO_4 (1 mL) was heated to reflux for 4 h then cooled and diluted with water (240 mL). The solution was extracted with diethyl ether (3 × 50 mL) and the combined organic extracts were washed with brine (2 × 50 mL), dried over $MgSO_4$ and concentrated *in vacuo* to give the title compound as a light-yellow oil (11.7 g, 99%).

^{1}H NMR (400 MHz, $CDCl_3$) δ 7.12 (d, *J* = 8.6, 2H), 6.74 (d, *J* = 8.6, 2H), 4.15 (q, *J* = 7.1, 2H), 3.54 (s, 2H), 1.25 (t, *J* = 7.1, 3H) **LRMS** (EI+) *m/z* 180 $[M]^+$

Ethyl 2-(4-((2-octyldodecyl)oxy)phenyl)acetate

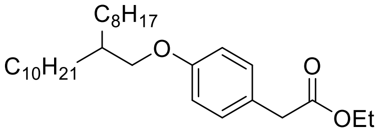

To a solution of ethyl 2-(4-hydroxyphenyl)acetate (11.6 g, 64 mmol) and 2-octyldodecyl bromide (29.7 g, 82 mmol) in anhydrous DMF (330 mL) under argon was added potassium carbonate (27.6 g, 0.2 mol). The reaction was heated at 80 °C for 12 h, then neutralised by the addition of 6M hydrochloric acid (20 mL). The suspension was filtered, and the filtrate was extracted with diethyl ether (3 × 100 mL). The combined organic extracts were washed with water (2 × 100 mL) then brine (2 × 100 mL), dried over $MgSO_4$ and concentrated *in vacuo*. The crude residue was purified by dry-flash chromatography eluting initially with hexane to remove excess 2-octyldodecyl bromide, then with 10% ethyl acetate in hexane to give the title compound as a pale-yellow oil (19.3 g, 64%).

^{1}H NMR (400 MHz, $CDCl_3$) δ 7.18 (d, *J* = 8.7, 2H), 6.85 (d, *J* = 8.7, 2H), 4.14 (q, *J* = 7.1, 2H), 3.80 (d, *J* = 5.7, 2H), 3.54 (s, 2H), 1.82 – 1.68 (m, 1H), 1.48 – 1.38 (m, 2H), 1.38 – 1.18 (m, 33H), 0.93 – 0.82 (m, 6H) **^{13}C NMR** (100 MHz, $CDCl_3$) δ 172.1, 158.6, 130.2, 125.9, 114.6, 70.9, 60.9, 40.6, 38.0, 32.0, 31.4, 30.1, 29.8, 29.7, 29.4, 26.9, 22.8, 22.7, 14.3, 14.2 **HRMS** Found (CI+): $[M]^+$ 460.3912, $C_{30}H_{52}O_3$ requires 460.3911

2-(4-((2-Octyldodecyl)oxy)phenyl)acetic acid

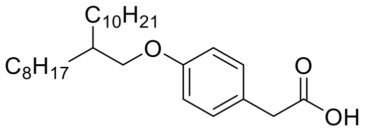

Under normal atmospheric conditions, aqueous sodium hydroxide (4M, 80 mL) was added to ethyl 2-(4-((2-octyldodecyl)oxy)phenyl)acetate (19.2 g, 42 mmol) in ethanol (80 mL) at RT. After 4 h the reaction was acidified, and the solution extracted with diethyl ether (3 × 100 mL). The combined organic extracts were washed with brine (2 × 50 mL), dried over $MgSO_4$ and concentrated *in vacuo* to give the title compound as a pale-yellow oil that solidified partially on standing (17.9 g, 99%).

^{1}H NMR (400 MHz, $CDCl_3$) δ 7.18 (d, *J* = 8.7, 2H), 6.85 (d, *J* = 8.7, 2H), 3.80 (d, *J* = 5.7, 2H), 3.58 (s, 2H), 1.81 – 1.70 (m, 1H), 1.48 – 1.38 (m, 2H), 1.38 – 1.19 (m, 31H), 0.88 (t, *J* = 6.8, 6H) **^{13}C NMR** (100 MHz, $CDCl_3$) δ 177.3, 158.8, 130.4, 125.0, 114.7, 70.9, 40.1, 38.0, 32.0, 31.4, 30.1, 29.8, 29.7, 29.4, 26.9, 22.8, 22.8, 14.2 **HRMS** Found (EI+): $[M]^+$ 432.3599, $C_{28}H_{48}O_3$ requires 432.3598

3,10-Dibromo-7,14-bis(4-((2-octyldodecyl)oxy)phenyl)diindolo[3,2,1-*de*:3',2',1'-*ij*][1,5]naphthyridine-6,13-dione

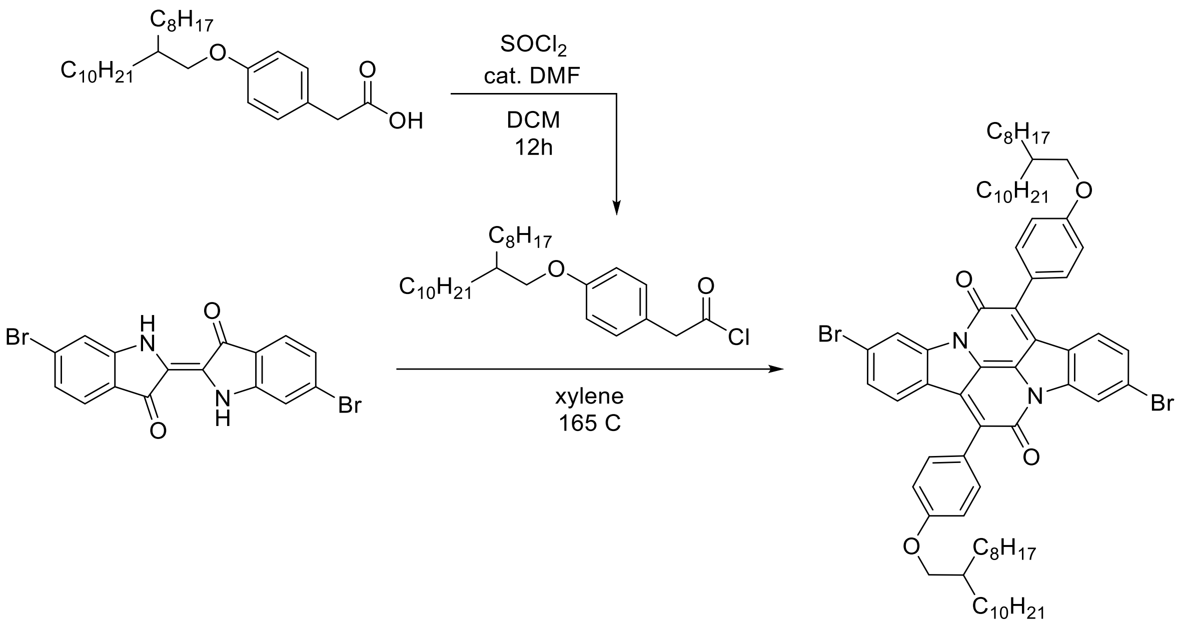

To a solution of 2-(4-((2-octyldodecyl)oxy)phenyl)acetic acid (24 g, 55.5 mmol) in anhydrous DCM (55 mL) was added catalytic anhydrous DMF (0.1 mL) and thionyl chloride (12.2 mL, 0.167 mol). The reaction was heated at reflux for 2 h and total conversion confirmed by NMR. All volatiles were carefully removed *in vacuo* to give the corresponding acyl chloride (**7**) quantitively which was used immediately without further purification.

To a suspension of 6,6'-dibromoindigo (5.8 g, 14 mmol) in xylenes (185 mL) was heated to 165 °C. Acyl chloride **2** (55.5 mmol) was added in one portion and the reaction heated for 24 h. Xylenes were removed *in vacuo* and the resulting crude residue purified by dry-flash chromatography eluting with 40% chloroform in hexanes. Fractions containing the product (R_F = 0.2, pink) were combined and concentrated *in vacuo* to give a residue which was then washed with ethyl acetate and the insoluble red solid collected by vacuum filtration (0.95 g, 6%).

^{1}H NMR (600 MHz, $CDCl_3$) δ 8.72 (d, *J* = 1.8 Hz, 2H), 7.65 (d, *J* = 8.8 Hz, 4H), 7.58 (d, *J* = 8.5 Hz, 2H), 7.37 (dd, *J* = 8.5, 1.8 Hz, 2H), 7.09 (d, *J* = 8.8 Hz, 4H), 3.96 (d, *J* = 5.6 Hz, 4H), 1.90 – 1.78 (m, 2H), 1.50 – 1.21 (m, 64H), 0.89 (td, *J* = 7.0, 5.3 Hz, 12H) **^{13}C NMR** (150 MHz, $CDCl_3$) δ 160.6, 159.6, 145.1, 131.7, 131.0, 130.6, 129.3, 126.3, 126.2, 125.1, 124.5, 122.1, 121.1, 114.7, 71.2, 38.1, 32.1, 31.5, 30.2, 29.8, 29.5, 27.0, 22.8, 14.3 **HRMS** Found (EI): $[MH]^+$ 1211.5818, $C_{72}H_{97}Br_2N_2O_4$ requires 1211.5815

1.1.2. DPPT synthesis

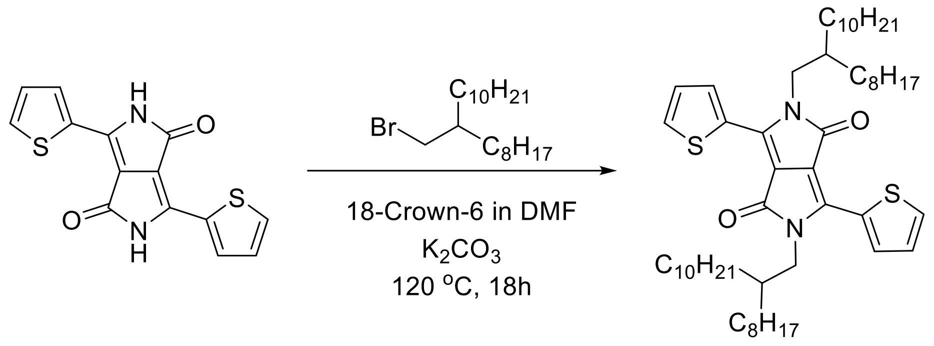

9 **10**

3,6-Di(thiophen-2-yl)-2,5-dihydropyrrolo[3,4-c]pyrrole-1,4-dione (9)

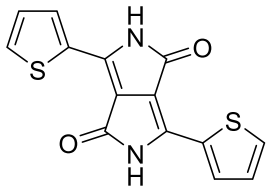

A flask containing sodium chunks (3.24 g, 140 mmol), 2-methyl-2-butanol (75 mL), and anhydrous iron (III) chloride (0.14 g, 0.863 mmol) was stirred at 150 °C under argon until all sodium was consumed (~30 min). The reaction was cooled to ~85 °C before thiophene-2-carbonitrile (8.5 mL, 91.3 mmol) was added, followed by diethylsuccinate (6.9 mL, 41.5 mmol). The mixture turned magenta in colour and was left to stir at 90 °C overnight. The mixture was allowed to cool to 50 °C and then methanol (122 mL) was added. The reaction was quenched with glacial acetic acid (36.6 mL) and refluxed for 45 min. The mixture was then filtered, washed with water (50 mL), acetone (50 mL), methanol (6 x 50 mL) and hexane (4 x 50 mL) and dried under vacuum to afford the product as a magenta waxy solid (7.1 g, 52 %) which was used without further purification.

^{1}H NMR (600 MHz, DMSO) δ (ppm): 11.26 (s, 2H, N*H*), 8.20 (s, 2H, Ar*H*), 7.96 (s, 2H, Ar*H*), 7.30 (s, 2H, Ar*H*. **^{13}C NMR** (600 MHz, DMSO): 161.7, 136.2, 132.8, 131.3, 130.8, 128.8, 108.6. **LRMS** (EI+) *m/z* 300 [M]$^+$.

2,5-Bis(2-octyldodecyl)-3,6-di(thiophen-2-yl)-2,5-dihydropyrrolo[3,4-c]pyrrole-1,4-dione (10)

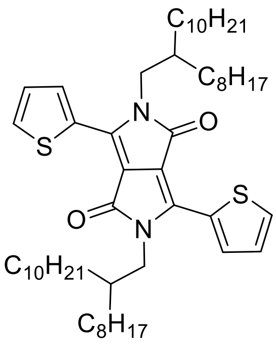

To a dry degassed flask under argon was added compound (**9**) (1.02 g, 3.4 mmol), 9-(bromomethyl)nonadecane (4.05 g, 11.2 mmol), potassium carbonate (1.55 g), 18-crown-6 (0.01 g), followed by anhydrous DMF (40 mL). The resulting magenta solution was heated to 120 °C and allowed to stir overnight. The reaction was cooled to room temperature and then concentrated *in vacuo*. The crude product was then purified *via* column chromatography on silica gel (hexane:chloroform = 4:1) to afford the product as a magenta solid (0.63 g, 22%).

^{1}H NMR (400 MHz $CDCl_3$) δ (ppm): 8.87 (dd, *J* = 3.9, 1.0 Hz, 2H, Ar*H*), 7.62 (dd, *J* = 5.0, 1.0, 2H Hz, Ar*H*), 7.28 (m, 2H, Ar*H*), 4.02 (d, *J* = 7.7, 4H, NCH_2), 1.90 (brs, 2H, NCH_2CH), 1.38-1.13 (m, 64H, CH_2), 0.89-0.84 (m, 12H, CH_3). **^{13}C NMR** (151 MHz, $CDCl_3$): δ 161.9, 140.6, 135.4, 130.6, 129.9, 128.5, 108.0, 46.3, 37.8, 32.0, 31.9, 31.3, 30.1, 29.8, 29.6, 29.4, 26.3, 26.3, 22.8, 22.8, 14.3. **LRMS** (EI+) *m/z* 749 $[M]^+$.

1.1.3. TIPS-tetracene synthesis

TIPS-tetracene was prepared as previously reported[1], and was purified by recrystallizing three times from hexanes.

1.2. Sample preparation

The diphenylanthracene blend solution was prepared in a nitrogen-filled glovebox with anhydrous toluene, at a diphenylanthracene concentration of 30 mg/mL and a diphenylanthracene:Pt-porphyrin:polystyrene ratio of 50:1:15 (wt/wt). Solution was heated at 90°C for 1 day and then filtered with a PTFE filter (400 µm). Films were spin-coated from hot solution. Solutions of DPPT and INDB were prepared in air, at a concentration of 6.25 mg/ml in toluene with an additional 25 mg/ml of polystyrene. To control for the effects of oxygen, DPPT solutions were also prepared in a nitrogen-filled glove-box with anhydrous toluene. TIPS-tetracene solutions were prepared in a nitrogen-filled glove-box with anhydrous toluene, at a concentration of 50 mg/ml. Thin films of all materials were spun on quartz-coated glass substrates for reference optical measurements.

To prepare microcavities, we first deposited a thick (150-200 nm) Ag mirror on quartz-coated glass substrates with a thermal evaporator. Films of the desired thickness (~200 nm, aiming for λ-mode microcavities) were spin-coated on top, within a glove-box in the case of oxygen-sensitive systems. We then evaporated the semitransparent top Ag mirror (25-30 nm) to complete the microcavities. Because of the high sensitivity of triplet-related dynamics to atmospheric oxygen[2], all samples were encapsulated prior to removal from the glovebox, with the exception of select DPPT films and microcavities used to study the oxygen dependence of our observations. We used the following protocol. Within a nitrogen-filled glovebox, films or microcavities were covered with a glass microscope coverslip of larger dimension than the substrate, using strips of 100 µm-thick carbon tape to prevent the surfaces from touching. We mixed a two-part fast-drying epoxy (Araldite) within the glovebox and applied this liberally around all four edges of the sample substrate, ensuring a complete seal with the coverslip. The samples were then allowed to dry for >18 hours prior to removal from the glovebox. Using this protocol, we find that samples retain their initial behaviour at least 1 year after fabrication, stored under ambient conditions.

Some reference samples were also prepared entirely within the thermal evaporator used for mirror deposition. These were prepared with either a 'thick' (73 nm) or 'thin' (20 nm) layer of TIPS-tetracene. For thick samples the organic layer consisted of a 20-nm layer of bathocuproine (BCP, Sigma Aldrich), the layer of TIPS-tetracene and a final 20-nm capping layer of BCP. BCP was evaporated at a rate of 0.3 Å/s, and TIPS-tetracene at a rate of 0.3 Å/s. For the 'thin' samples the organic layer consisted of a 90-nm layer of BCP, a layer of TIPS-tetracene of 20 nm and a final 90 nm capping layer of BCP. BCP was evaporated at a rate of 1 Å/s for these samples, and TIPS-tetracene at a rate of 0.3 Å/s. Mirror deposition and encapsulation were performed identically to spin-cast samples.

1.3. Steady-state measurements (absorption, PL, reflectivity)

The absorption and photoluminescence spectra of reference films were acquired with a Fluoromax spectrofluorometer (Horiba Jobin Yvon) equipped with the F3031 transmission accessory. Together with the film thickness determined using a Dektak stylus profiler, these spectra were used to determine the optical parameters for transfer matrix modelling. Microcavity reflectivity maps were obtained on a home-built goniometer system, using motorised arms for excitation and collection that allow the angle of incidence to be swept from 10° to 60°. White light was provided by a fibre-coupled lamp with deuterium and halogen sources. Collected light was sent via fibre to an Andor Shamrock CCD spectrometer. Steady-state photoluminescence spectra were obtained with the time-resolved photoluminescence system (below), using sufficiently long gate windows to capture the full microcavity or film dynamics.

1.4. Time-resolved photoluminescence

Time-resolved photoluminescence measurements were acquired with an Andor iStar ICCD, coupled to a Shamrock 303i spectrograph. Excitation was provided by an Nd:YAG Q-switched laser (Innolas Picolo) tuned for second-harmonic (532 nm) or third-harmonic (355 nm) output and synchronized with the ICCD. Nominal pulse duration is 600 ps, at a typical repetition rate of 5 kHz. Excitation and collection were through the same aspheric condenser lens (f=3.2 mm, NA=0.76), with the excitation incident on the sample at 40°. Angle-dependent measurements were performed using an additional k-space lens to image the rear Fourier plane of the excitation/collection optic.

Two of the materials systems studied – DPA:PtOEP blends and DPPT – exhibit delayed fluorescence through bimolecular triplet-triplet annihilation. To be able to compare between thin-film and microcavity measurements, it is thus essential to correctly account for differences in excitation density caused by the mirrors of the microcavity. These considerations do not apply to TIPS-tetracene, where all of the relevant processes are geminate. For the bimolecular systems, we use simple optical considerations to determine the appropriate excitation scaling factor for microcavity vs film. We account for partial transmission through the semitransparent top mirror and multiple passes of the light through the film due to internal reflections, attenuated by the organic dye absorption. For example, in the DPA:PtOEP microcavities the 532 nm excitation is partially transmitted through the top mirror (~17%), and losses through this mirror on internal reflection constitute the main depletion channel of the pulse. Because of the weak absorption of the dilute porphyrin layer, internal reflections yield only 6.6% absorption of the incident light at 532 nm, compared with 9.8% in the mirror-backed films measured for comparison. Therefore, in our comparison of film samples measured at 50 µW incident laser power and microcavities measured at 150 µW in Figure 2d in the main text, the microcavities have a higher excitation density (~2x). As shown in Fig S3b below, in these systems this typically results in a faster decay of delayed fluorescence. The kinetic enhancement we report in main-text Figure 2d is thus despite the higher intrinsic triplet depletion rate.

1.5. Transfer matrix simulations

Microcavity reflectivity data was modelled using transfer matrix simulations as previously reported[3], based on the measured absorption of the organic films. The absorption spectra were fitted to a series of Lorentzian peaks, with the oscillator strength of each individually tuned to correctly reproduce the extinction of the measured film. A uniform film of these absorbers was then modelled between two Ag mirrors (200 nm and 30 nm), using tabulated value for the Ag index of refraction. The optical properties of the entire system (transmission, absorption, reflectivity) were calculated as a function of angle for comparison to the measured reflectivity maps. For these purposes, the index of the refraction of the organic layer inside the microcavity was treated as a free parameter, and it was adjusted to provide the correct dispersion for uncoupled cavity modes observable at shorter and longer wavelengths than the exciton-polariton bands of interest. Good fits were obtained with typical values ~1.6. The oscillator strength of the organic film was also adjusted slightly compared to the bare-film reference, to allow for slight variation in dye concentration from batch to batch, spatial inhomogeneity (reflectivity measurements are taken over a ~0.5 mm spot, versus 8 mm aperture for absorption) and inevitable partial sample degradation in air for reference film measurements (due to the need to subsequently measure film thickness). In all materials, an adequately parametrized model which closely describes the film absorption can also closely reproduce the measured reflectivity maps, confirming that all samples are within the strong exciton-photon coupling regime. In addition to the peak positions of the polariton branches which are output by the transfer matrix model, we also include the input exciton peak positions and cavity photon mode dispersions as dashed lines in the main-text figures and Figures S3 and S7 below.

2. Diphenylanthracene:Pt-porphyrin:polystyrene 50:1:15

Figure S1 shows the steady-state absorption and emission from the blend film. The vibronic progression of diphenylantrhacene can be clearly distinguished, while the absorption 500-550 nm of the porphyrin dopant is markedly weaker. At this low loading the porphyrin is too weakly absorbing to enter the strong-coupling regime itself. Following excitation at 355 nm (blue), we measure direct diphenylanthracene fluorescence 400-500 nm, as well as substantial Pt-porphyrin phosphorescence due to rapid energy transfer from dipyhenylanthracene and intersystem crossing[4,5]. Exciting Pt-PEOP results in up-converted emission at 400-450nm from diphenylanthracene. As this is a solid-state blend, phosphorescence at 650nm dominates the spectrum due to significant phase-separation, which prevents Pt-PEOP triplets from reaching the diphenylanthracene regions of the film[4,5].

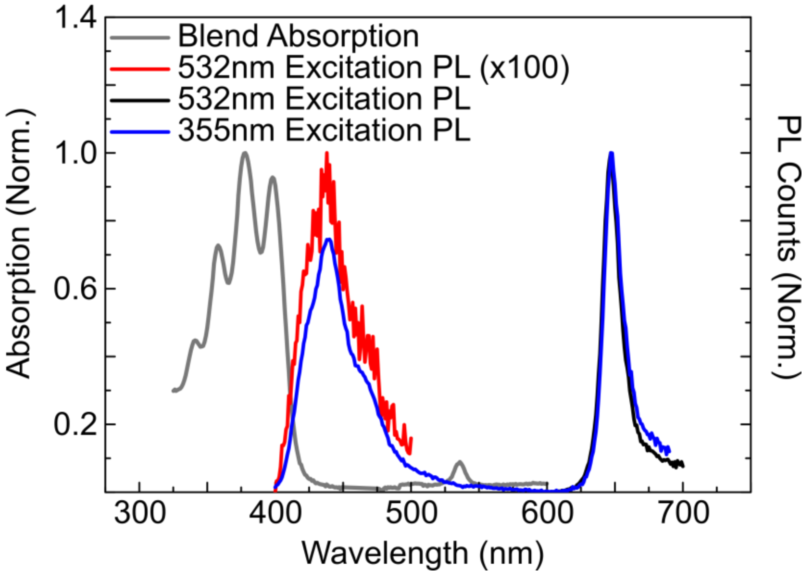

Figure S1. Steady-state characterisation of photon up-conversion blend. Absorption spectrum (grey) of blend film, and photoluminescence spectra following excitation at 350-355 nm (blue) and at 530 -535 nm (black). As indicated, the diphenylanthracene emission following excitation at 530-535 nm is scaled 100x (red).

In Figure S2 we show the delayed emission decay kinetics for the cavity at three excitation powers. As we increase the power, we see a reduction in the PL lifetime consistent with the well-known bimolecular process of triplet-triplet annihilation. As described in Section 1.4 above, we account for this intensity dependence when we compare measurements taken with 50 µW incident laser power for the film samples with 150 µW for the microcavities in Figure 2d in the main text.

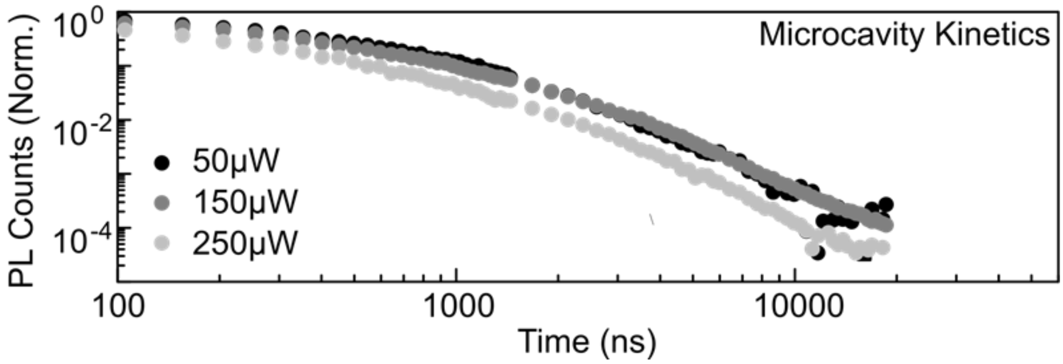

Figure S2. Excitation power dependence of diphenylanthracene:PtOEP blend microcavity emission, exhibiting faster decay at higher powers consistent with bimolecular triplet-triplet annihilation within diphenylanthracene domains.

337

3. Indolonaphthyridine benzene (INDB) – Triplet-free reference

To investigate the importance of triplet-triplet annihilation to our observed polaritonic enhancement, we performed similar studies on a dye which exhibits no observable delayed emission and shows no evidence of triplet formation: indolonaphthyridine benzene (INDB). Figure S3 shows a reflectivity map of a Ag-Ag microcavity containing an INDB blend film. The clear anti-crossing at the 0-0 absorption energy and transfer matrix modelling (lines) confirm that this microcavity is in the strong-coupling regime. Following photoexcitation of the microcavity, all emission is from the lower polariton branch.

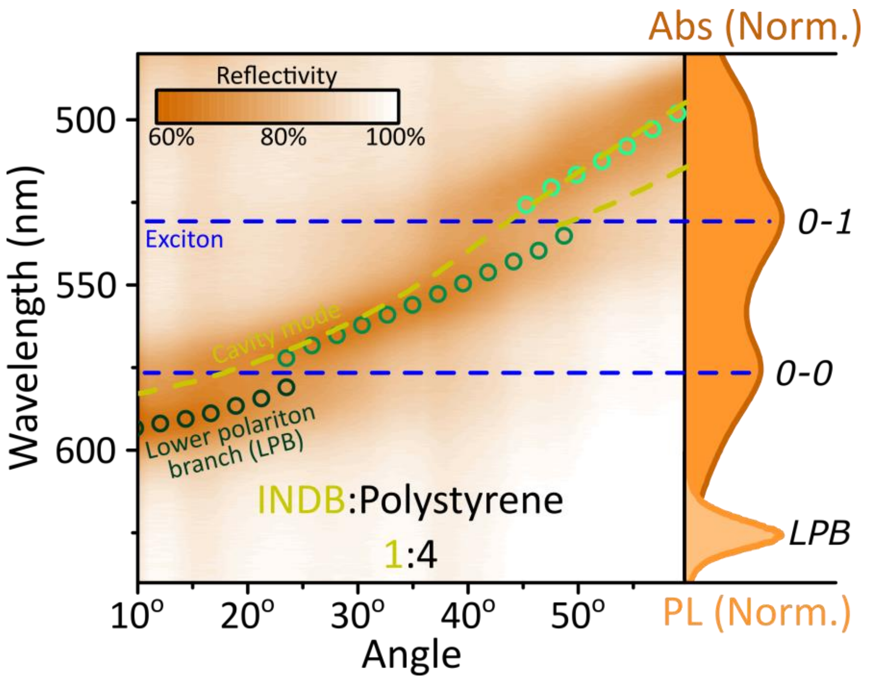

Figure S3. Reflectivity map of INDB microcavity. Ag-Ag microcavity containing INDB:polystyrene (1:4 by weight) film. Comparison with absorption spectrum (right) and transfer matrix modelling (lines, circles) confirms strong coupling. All emission arises from the lower polariton branch (LPB).

INDB film and microcavity emission dynamics are shown in Figure S4, integrated over the entire spectral range (i.e. both singlet and excimer bands in the film). In the thin film, beyond the instrument response region the emission is dominated by a red-shifted, featureless excimer band, as is typical for such planar molecules. Kinetics for the film and microcavity samples are similar, confirming the excimer can also populate the lower polariton branch. This effect has been previously reported in microcavities containing a BODIPY dye and described through a 'radiative pumping' mechanism[6]. Because the parent state in this process is by necessity already emissive, the microcavity has no substantial effect on the emission lifetime between cavity and film samples. There is no detectable enhancement in 'delayed' emission. These measurements confirm that the large changes in lifetime we report in the main text are not an artefact of our measurement conditions. In the absence of triplet-triplet annihilation, we observe no significant lifetime enhancement between film and microcavity.

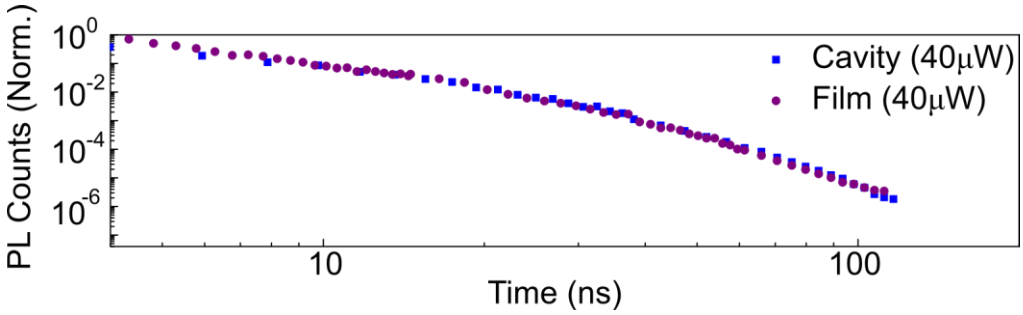

Figure S4. INDB emission kinetics. Emission kinetics integrated over full spectral bandwidth of bare film (circles) and microcavity (squares) of INDB.

4. TIPS-tetracene

Because the effects of strong coupling are most pronounced in this material, we performed additional processing checks on TIPS-tetracene films to ensure our observations are not related to materials handling. The full results are tabulated in Table S1, with detailed spectra and kinetics in the following sections.

Table S1. Summary of sample types and observed behaviour.

Prep. no.	Sample type	Strong coupling?	Strong delayed PL?	Notes
**1**	Spin-cast thin film (~200 nm) on glass, toluene solution	*no*	*no*	reproduces literature behaviour
**2**	Spin-cast thin film (~200 nm) on glass, chloroform solution	*no*	*no*	change in morphology does not alter dominant PL dynamics
**3**	Spin-cast thin film (~200 nm) on glass, toluene solution, thermally annealed	*no*	*no*	improves morphology and reduces disorder but only slightly enhances delayed emission
**4**	Spin-cast thin film (~200 nm) on glass, toluene solution, exposed to ultrahigh vacuum	*no*	*no*	removes residual solvent and possible quenching sites, improves morphology but dynamic effects are within sample to sample fluctuation
**5**	Spin-cast thin film (~200 nm) on Ag, toluene solution	*no*	*no*	no effect, reproduces reference film within sample-to -sample variance. Ag promixity does not result in any enhancement
**6**	Evaporated TIPS-tetracene film on glass	*no*	*no*	reproduces spin-cast film behaviour
**7**	Evaporated BCP:TIPS-tetracene:BCP film (20 nm:73 nm:20 nm) on glass	*no*	*no*	slight change in spectral shape due to increased disorder at TIPS-tetracene:BCP interface, but evaporation with BCP does not significantly alter photophysical properties
**8**	Evaporated BCP:TIPS-tetracene:BCP film (20 nm:73 nm:20 nm) on glass, capped with 25 nm Ag	*no*	*no*	minor effect, similar to thermal annealing or ultrahigh vacuum treatment. Microcavity processing procedures are insufficient to cause enhanced delayed emission in the absence of strong coupling
**9**	Evaporated BCP:TIPS-tetracene:BCP film (90 nm:20 nm:90 nm) on glass	*no*	*no*	no effect, photophysics of thin film are identical to thick films
**10**	Evaporated BCP:TIPS-tetracene:BCP film (90 nm:20 nm:90 nm) on Ag, capped with 25 nm Ag	*no*	*no*	no effect, encapsulation within a non-coupled microcavity does not enhance the delayed emission
**11**	Spin-cast thin film (variable thickness) on Ag, toluene solution, capped with 25 nm Ag	**yes**	**yes**	polariton formation results in substantially enhanced delayed emission
**12**	Evaporated BCP:TIPS-tetracene:BCP film (20 nm:73 nm:20 nm) on Ag, capped with 25 nm Ag	**yes**	**yes**	polariton formation results in substantially enhanced delayed emission, even when there is no physical contact between active layer and Ag

4.1. Solution-processed control samples

Spin-cast control films (2) were prepared from chloroform solutions as in the main text, to determine if the slight changes in morphology that result[7] have any effect on the delayed emission dynamics. Films types (3) and (4) were prepared identically to those in the main text, and then subjected to thermal annealing or ultrahigh vacuum in the evaporation chamber. These measurements allowed control for whether any incidental effects of microcavity preparation (e.g. changes in morphology under vacuum) could be the origin of the enhanced delayed emission reported in the main text. Other control samples were prepared using the same solution processing protocol on Ag mirrors (5). All samples were encapsulated and measured following the same procedures used for all other samples. We observe no systematic change in the photoluminescence decay kinetics following these solution-based processing steps, as summarised in Figure S5. Even for films with no additional treatments, we observe slight variation in the PL lifetime (panel a). Similar variation can be obtained by scanning across the same film, and can be attributed to slight changes in the crystallinity, resulting in a better-defined and more uniform cut-off for the initial S_1-^1TT equilibrium. Following annealing, vacuum treatment or deposition on a mirror we observe variation on a similar scale (panel b), in some cases with increased prominence of the ‘kink’ as the kinetics transition from cleanly exponential behaviour to a less-defined power-law-type decay weakly detectable above the noise floor. In no instance do we detect changes in the film behaviour commensurate with the effects of microcavity formation, confirming that the effects we report in the main text do not arise from our processing or metal-organic interactions, but are instead uniquely caused by strong light-matter coupling.

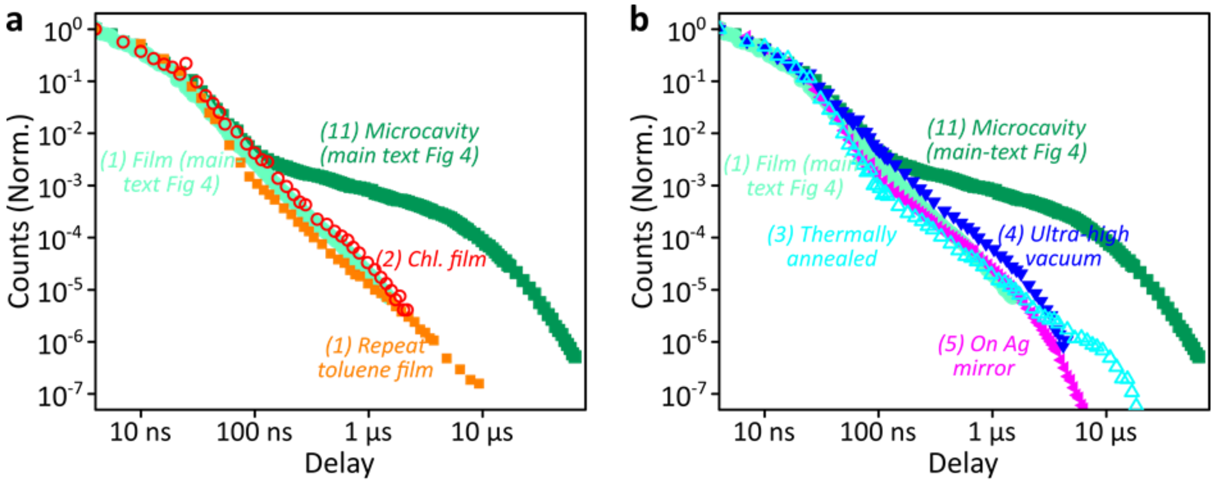

Figure S5. Control measurements on TIPS-tetracene films. **a** Films prepared from different batches of solution or different solvent at the same TIPS-tetracene exhibit the same qualitative behaviour. The slight spread of lifetimes indicates slight variations in film crystallinity; similar variation can be obtained scanning within the same film. **b** Exposure of the films to heat or ultra-high vacuum does not significantly change the kinetics beyond the standard film-to-film variability. Similarly, deposition on an Ag mirror does not result in a major change in lifetime due to metal-organic interactions. No processing steps result in an enhancement that can explain the behaviour observed in full microcavities. In each kinetic only every third data point is shown for clarity.

4.2. Fully evaporated control samples

We additionally prepared fully evaporated sample structures in which the TIPS-tetracene active layer is surrounded by two 20-nm spacer layers of BCP, to ensure that direct physical contact between TIPS-tetracene and Ag is not responsible for the observed delayed emission. Samples in this section are described with reference to the preparation number in Table S1. We first confirmed that evaporation of TIPS-tetracene does not yield films with significantly different emission properties (Figures S6, S8a). Multilayer BCP:TIPS-tetracene:BCP films (7) and (9) on glass exhibit a redshift of the overall PL spectral weight, consistent with a minor contribution from excimer-type sites previously reported to exist in disordered samples[8]. These features do not dominate the emission and are presumably a minority species in the film, and we expect they are formed at the TIPS-tetracene:BCP interface where disorder will be greater. There is no accompanying change in the PL decay dynamics: evaporated and spin-coated samples are fully equivalent. Likewise, subsequent coating (8) with an evaporated Ag mirror does not significantly enhance the delayed emission and results in changes comparable with film-to-film variation.

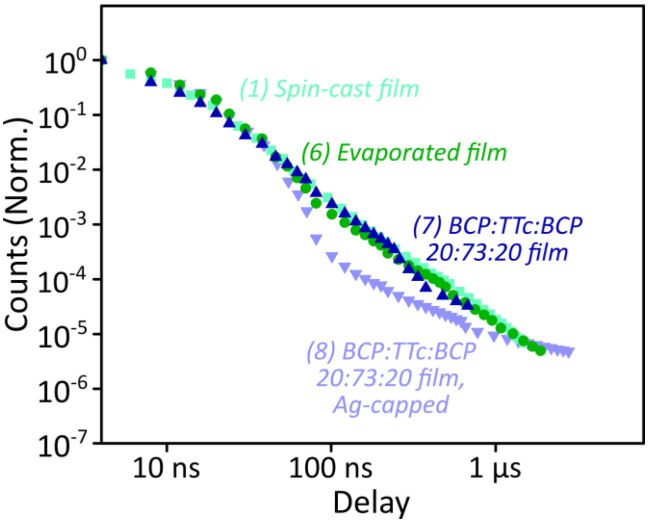

Figure S6. Evaporation of TIPS-tetracene results in the same photoluminescence dynamics, whether in a pure film or a multilayer structure with BCP. Subsequent evaporation of a Ag capping layer does not substantially alter the delayed emission, with similar effects to thermal annealing or vacuum treatment (see above), and it offers no enhancement.

Sample (10) is a full microcavity structure, with a sufficiently thin layer of TIPS-tetracene and a sufficiently large negative detuning that no strong light-matter coupling is possible. This sample exhibits effectively identical dynamics to other reference films, demonstrating that the full microcavity processing steps are insufficient to induce major enhancement to the delayed emission if polariton formation is not possible. However, when we prepare full microcavities with a thick evaporated layer of TIPS-tetracene (12) we observe clear strong light-matter coupling in the angle-dependent reflectivity (Figure S7), well-defined polariton emission (Figure S8a) and the same substantial enhancement to delayed emission (Figure S8b) reported in the main text for spin-cast microcavities (11). The enhancement is clearly

independent of physical contact between the TIPS-tetracene and metal mirrors or any of the sample processing steps, and it is thus a unique consequence of polariton formation.

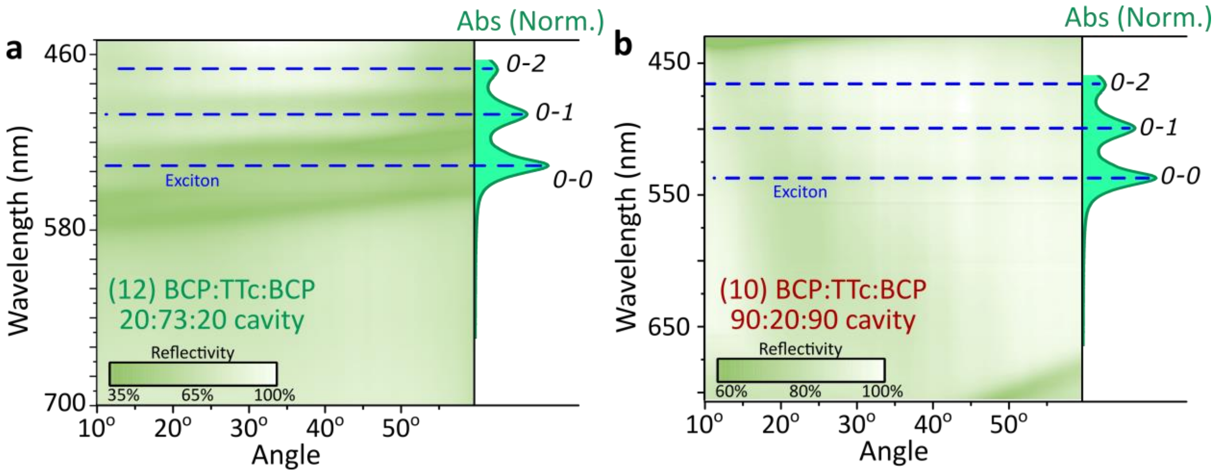

Figure S7. Fully evaporated TIPS-tetracene samples. **a** Angle-dependent reflectivity of the ‘thick’ TIPS-tetracene evaporated cavity shows strong coupling as in solution-processed cavities. **b** No strong coupling is evident in the ‘thin’ TIPS-tetracene evaporated cavity.

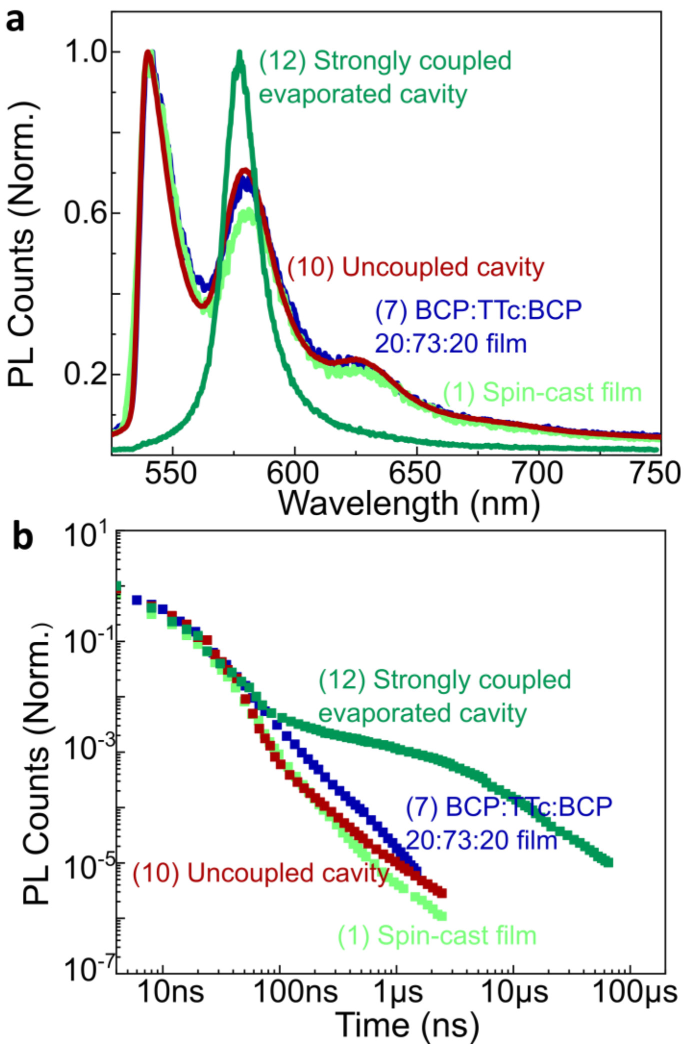

Figure S8. Characterisation of fully evaporated samples. **a** Emission spectra and **b** integrated kinetics of each sample. Data is acquired and processed as in main-text Figure 3. Comparison of samples produced by evaporation and spin casting shows little variance. A strongly detuned microcavity with low oscillator strength (‘Uncoupled cavity’) also exhibits no enhancement in the delayed emission.

4.3. Detuning dependence

We have also measured the dependence of the observed enhancement on the energy offset between cavity mode and S_1 (the ‘detuning’). Figure S9 shows that across the entire detuning series we observe the same qualitative effect, though the magnitude and lifetime of the enhancement appears to vary. There is no systematic dependence on energy offset. Interestingly, the least and most negatively detuned cavities (-71 and -545 meV) exhibit nearly the same long-time kinetic. The extremes of dynamic behaviour are instead obtained with two similar ‘intermediate’ negative detunings, -342 and -418 meV. The fairly short energy scale for such changes in behaviour is consistent with earlier studies of squaraine microcavities[9], where the detuning scans the LPB over relatively sharp energetic resonances

within the exciton reservoir. The complex structure observed here suggests the presence of multiple resonances, likely related to the more complex vibronic structure of TIPS-tetracene.

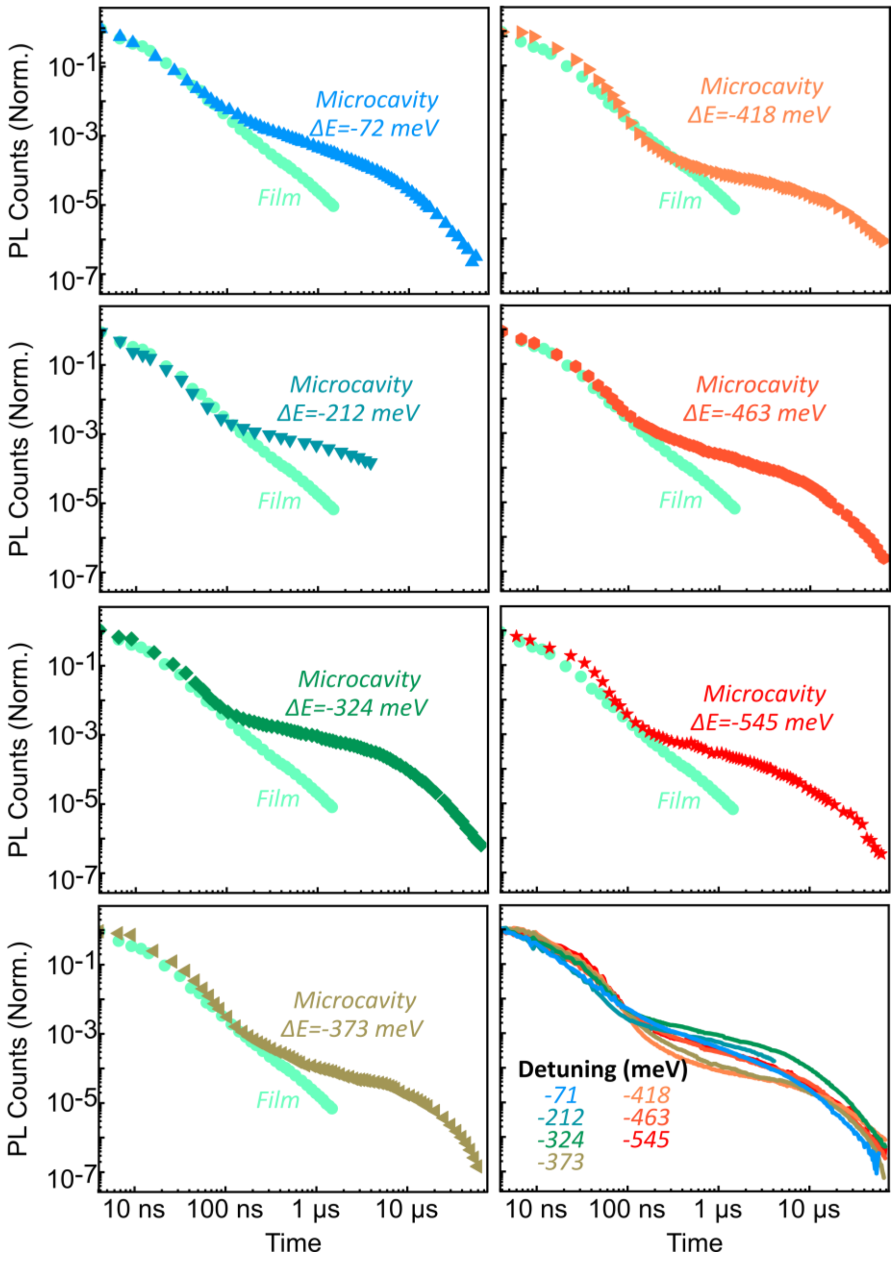

Figure S9. Detuning dependence in TIPS-tetracene microcavities. Integrated PL kinetics, acquired and processed as in main-text Figure 3. The same qualitative behaviour is observed over the full range, but there is no systematic dependence of the relative magnitude or lifetime of the enhanced emission. In each kinetic only every fifth data point is shown for clarity.

In the detuning-dependent kinetics above, the microcavity emission was collected with the same NA=0.76 lens used for other measurements and thus effectively integrated across the entire LPB. The spectral shape revealed that emission was predominantly from near the bottom of the branch, at 0°. In addition, we measured the angular dispersion of steady-state emission for selected cavities using a goniometer system able to measure up to 70°. We

corrected these dispersions for the photon fraction along the LPB to determine the polariton population distributions as a function of detuning, presented in Figure S10. We make two primary observations. 1) The population for every detuning is predominantly located at the bottom of the LPB, suggesting the pathway to reach the branch bottom must not depend strongly on energy offset. Given the short lifetime (<<100 fs) of individual polaritons within our low-Q (30-60) cavities, it is very unlikely these states are populated through polariton-polariton scattering or vibrational relaxation along the LPB. Instead, as is typical for such organic microcavities dominated by reservoir dynamics[6,10], the distribution of polariton population relates directly to the distribution of parent states, taking into account that the pathway from reservoir to LPB may release energy. In light of restrictions on the energy of the parent $^{1/5}$TT states in the reservoir (the TT states are expected to be very close in energy to S_1, not deeply stabilised), this result is surprising. It points to a wide, effectively continuous distribution of parent reservoir states, of accepting LPB states (for instance, higher vibronic polaritons that emit at the same energy as the LPB[11,12]) and/or of vibrational scattering channels enabling resonant transfer to the LPB bottom. 2) The cavities show slight enhancement in the populations at energies ~1.9 eV and ~2.1 eV, regardless of the angle at which this energy occurs. This distinctive resonance effect would be approximately consistent with radiative pumping from $^{1/5}$TT, if these peaks are taken to be the 0-1 and 0-2 vibronic replicas (0-0 origin ~2.25 eV). Alternatively, it could be consistent with a model in which vibronic polariton branches above the LPB[11,12] are brought into resonance with the $^{1/5}$TT reservoir, resulting in more efficient transfer.

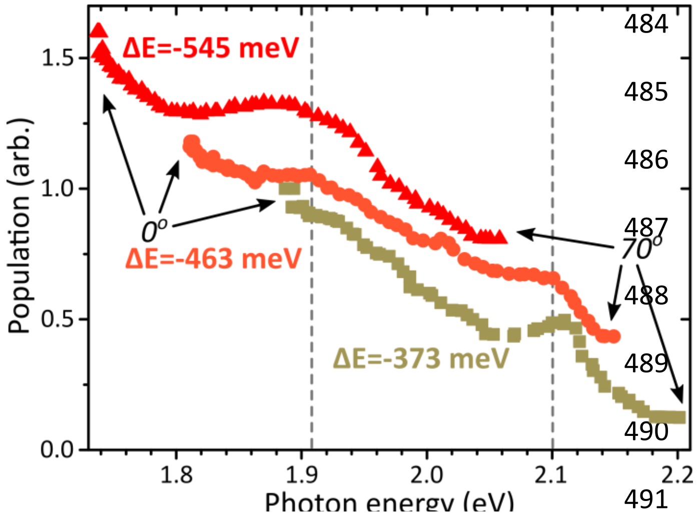

Figure S10. LPB population distribution. Angle-dependent emission from three negatively-detuned TIPS-tetracene microcavities, corrected for photon fraction to yield population. Distinct enhancement is detected whenever the LPB crosses ~1.9 eV and ~2.1 eV (dashed lines). The 0-0 exciton peak which forms the polaritons is located at ~2.3 eV. Regardless of the resonant enhancement, the polariton population is predominantly located at or near the bottom of the LPB for every detuning. Plots are vertically offset for clarity.

4.4. Spectral/k-space evolution

Both in films and microcavities of TIPS-tetracene, we observe that the spectral shape does not change over the entire decay range. We show this in Figure S11 with spectra taken within the instrument response and at very long time delay. We observed similar behaviour for microcavities of all materials (including in time-resolved k-space measurements), and films of the photon up-conversion blend. In all instances, this demonstrates that the emission process is mediated by the same state throughout the decay lifetime. In TIPS-tetracene films, this is the S_1 state (whether directly photogenerated or regenerated from triplet-triplet annihilation). In microcavities, the emission is always from the lower polariton branch.

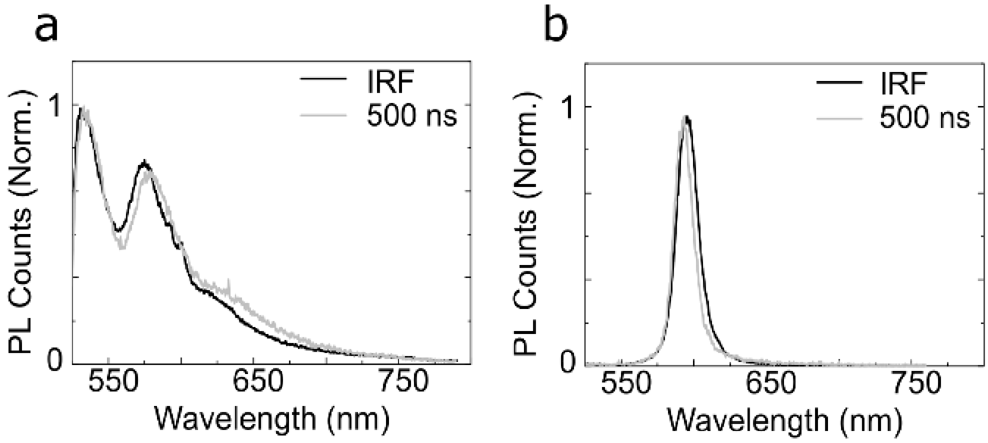

Figure S11. Time-resolved TIPS-tetracene emission. **a** Spectra of pure TIPS-tetracene film following excitation at 532 nm, collected within the instrument response (IRF) and at 500-600 ns after excitation. **b** Equivalent spectra for microcavity.

5. Rate modeling

To understand the mechanism of ultra-long-lived polariton emission, we have constructed a basic toy rate model for the TIPS-tetracene data. In principle this model could be extended to cover triplet-triplet annihilation in the other systems in the main text, but as this would involve dealing with exciton migration in a disordered and complex density of states, it is beyond the current scope of the work. The model, shown in Figure S12, is based on the current photophysical model of polycrystalline films of TIPS-tetracene presented in Weiss et al.[13] and Stern et al.[8] In this model singlet fission from S_1 (k_{SF}) creates a population of spin-entangled triplet-pairs ^{1}(TT), given by:

$$\left|{}^1TT\right\rangle = 3^{-1/2}(|xx\rangle + |yy\rangle + |zz\rangle) \qquad (1)$$

where $|xx\rangle$, $|yy\rangle$ and $|zz\rangle$ are the zero-field triplet-pair basis states. ^{1}(TT) is not an eigenstate of the spin-Hamiltonian in the regime of weak exchange coupling. In our model we term ^{1}(TT) 'TT_{bright}' as this population is in equilibrium with S_1 through k_{-SF} and is observed through delayed emission from S_1. Over ~10ns, the ^{1}(TT) states lose their spin-entanglement, and probably some S_1 character, and evolve into dark triplet pair states, 'TT_{dark}'. As there have been no spin-flips, the triplet pairs are still made up of $|xx\rangle$, $|yy\rangle$ and $|zz\rangle$, and are initially weakly exchange coupled with mixed singlet ($\left|{}^1TT\right\rangle = 3^{-1/2}(|xx\rangle + |yy\rangle + |zz\rangle)$) and quintet [$\left|{}^5TT_a\right\rangle = 2^{-1/2}(|xx\rangle - |yy\rangle)$ or $\left|{}^5TT_b\right\rangle = 6^{-1/2}(|xx\rangle + |yy\rangle - 2|zz\rangle)$] character. In the weakly exchanged-coupled regime the individual triplets that make up the pair have only weak wavefunction overlap.

These weakly-coupled TT states are thought to co-exist with a population of strongly exchange coupled triplet-pairs[13]. With strong exchange coupling, the triplet-pair states are pure singlet (S=0) or quintet (S=2) states as S becomes a good quantum number in the strong-exchange coupling regime. The latter have been observed in TIPS-tetracene films using transient EPR[13]. The evolution between the initially created weakly exchange-coupled and the observable strongly exchange-coupled triplet-pair states most likely occurs via dynamic off-diagonal disorder[13]: thermal fluctuations that alter the relative spacing between molecules and thus the wavefunction overlap between them. This has been described in similar systems by Troisi *et al.*[14]

If these fluctuations are responsible for generating strongly exchange-coupled triplet pairs, we can assume an equilibrium exists between weakly and strongly exchange-coupled TT states. Therefore, the dark triplet-pair population TT_{dark} (taken to be the equilibrium combination of weakly- and strongly-coupled singlet/quintet TT states) should follow the observed EPR quintet dynamics. We note that EPR lifetimes do not necessarily represent the natural lifetimes of the system, which is perturbed by the EPR measurement itself. The natural 'TT_{dark}' lifetime could be longer than the EPR-measured quintet lifetime.

Nevertheless, we take the EPR-measured quintet lifetime as a reasonable estimate of the TT_{dark} lifetime.

TT_{dark} represents the equilibrium population of weakly exchange-coupled TT states with mixed singlet/quintet character and strongly exchange-coupled pure quintet states. Eventually spin-flips can occur, probably through spin-orbit coupling, effectively scrambling the spins within the triplet-pairs. At this point the zero-field basis states include combinations such as $|xy\rangle$, $|xz\rangle$, etc. In this form, instead of all states (in the weakly exchange-coupled regime) showing some singlet character (i.e. containing $|xx\rangle$, $|yy\rangle$ or $|zz\rangle$), only 3/9 now have any singlet character. In this regime the triplets, although possibly still spatially bound in TIPS-tetracene as suggested by transient absorption measurements[8], are no longer correlated ('T_1+T_1'). There is no signature of quintet pairs in transient EPR at this point, but their excited-state absorption can still be measured using transient absorption spectroscopy[8] and transient EPR spectra[13] show evidence of weakly exchange-coupled triplets. It is likely that some of the triplet pairs have fully dissociated on this timescale, forming spatially separated uncorrelated triplets, but we assume this is a negligible process in TIPS-tetracene.

In TIPS-tetracene-based microcavities we assume that the lower polariton branch dynamics are governed by the exciton reservoir photophysics. Therefore, we use photophysical data from the literature to parameterise our model. The rate constants from our model are shown in Table S2 and comparison of our model to literature data is shown in Figure S12b. Our basic model – in the absence of strong light-matter coupling – fits all three observables: delayed fluorescence, transient absorption (TA) and transient EPR. We have attempted to constrain the model by using the fewest free parameters per population, fixing the radiative, singlet fission and fusion rates from literature values and the intrinsic non-radiative rates for S_1 and TT_{bright} from estimates based on the gap-law of non-radiative decay. The gap-law has been shown to be a good predictor of non-radiative rates for acene and hetero-acene-type materials[15]. The free parameters are: k_{dark}, which is fixed by fitting to the S_1 delayed emission; k_{nr} and k_{spin} of TT_{dark}, fixed by fitting to the tr-EPR and TA data; and k_{nr} of T_1+T_1, fixed by fitting to the terminal decay in the transient absorption data. Only k_{nr} and k_{spin} of TT_{dark} are not independently varying parameters. These are difficult to decouple as not enough is known about the intrinsic non-radiative decay of unentangled triplet-pairs or the time-constant for the formation of uncorrelated triplet pairs.

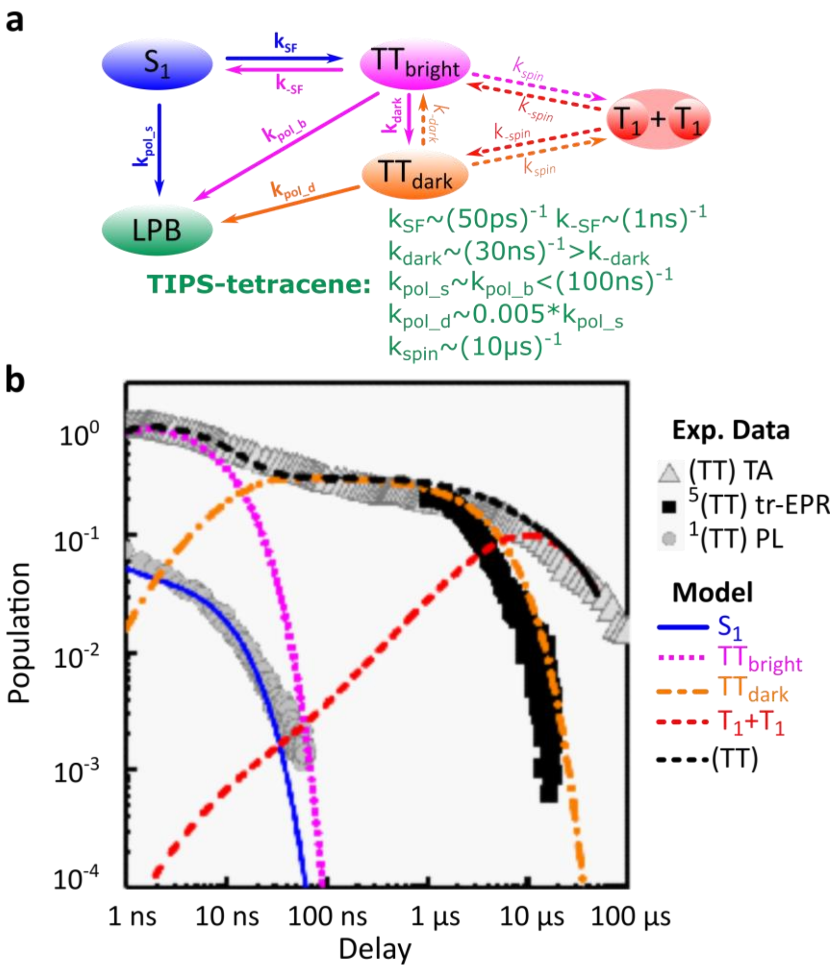

Figure S12. Basic rate model used to model TIPS-Tetracene film and microcavity emission. **a** schematic of rate model, all rates are reproduced in Table S2. Parameters are set from literature or fit to literature data[8,13,16]. **b** Comparison of our basic rate model with published optical (TA and PL)[8] and spin-resonance spectroscopy[13]. Grey circles indicate delayed emission from S_1, which tracks our S_1 population (blue line) and TT_{bright} population (pink dotted line) as S_1 and TT_{bright} are in equilibrium. Black squares indicate observed quintet dynamics from EPR. We fit our TT_{dark} population to match this decay (orange dot-dashed line). Grey triangles show transient absorption of triplet-pair states which is proportional to the sum of all triplet-pair populations. In our model (TT) = TT_{bright} + TT_{dark} + T_1+T_1, given by the black dashed line. We fit the T_1+T_1 lifetime to the decay of the transient absorption signal.

Table S2. Rates used in the rate model described in Figure S12 with results shown in Figures S13-15.

RATE CONSTANT	VALUE	REFERENCE OR RATIONALE
**G**	1/(0.5ns)	Generation estimated from instrument response.
**LPB K_R**	1/(0.1ns)	Should be roughly equal to the photon lifetime (here we make it longer to speed up computation time, noting that it ismarkedly faster than other rates on our measurement timescales).
**S_1 K_R**	1/(15ns)	Singlet radiative lifetime in solution[17]
**S_1 K_{NR}**	1/(30ns)	Assuming the non-radiative decay is dominated by the gap-law, as for other acenes, with E_{S1} = 2.3 eV [15]
**TT_{BRIGHT} K_R**	1/40 x S_1 k_r	Relationship previously determined for a similar heteroacene[18]
**TT_{BRIGHT} K_{NR}**	1/15	Assuming ^{1}(TT) decays via the same non-radiative gap-law as S_1, as demonstrated by the authors in an up-coming review. Energy = 2xT_1 = 2.2.eV.
**TT_{DARK} K_{NR}**	1/5000	Fit to the published ^{5}(TT) data[13]. Note this constant is not unique but is correlated with k_{spin} below.
**TT_{UNCORR} K_{NR}**	1/30,000	Fit to the transient absorption data[8].
**K_{SF}**	1/0.05	50ps[8]
**K_{-SF}**	1/1	Taken from a previous determination for tetracene[16]
**K_{POL_S}**	1/100	Fit to data (this is a maximum rate, any faster and the LPB emission would decay faster than we observe. Smaller values are possible but do not change the dynamics, only the relative LPB population).
**K_{POL_B}**	1/100	Fit to data (as above)
**K_{POL_D}**	x * k_{pol_s}	Fit to data (see Figure S14). For data in the main text x = 0.005.
**K_{DARK}**	1/30	Fit to delayed emission[8]
**K_{-DARK}**	0	In simplest model. Otherwise we used a distribution of rates to model the non-exponential tail of S_1 emission, see Figure S13.
**K_{SPIN}**	1/10,000	Fit to ^{5}(TT) EPR data[13] (see note for K_{NR} TT_{dark} above).

Having parameterised the model using reference data, we modelled our own data. Using all the same parameters as those fit to reference data in Figure S12, we first modelled our delayed fluorescence, Figure S13. The basic model (solid black line 'S_1') reproduces the initial exponential decay, as expected, but does not reproduce the power-law-like tail. To account for this tail, we adapted the model to assume a distribution of k_{-dark} from $(30ns)^{-1}$ to smaller rate values. Using this distribution (dashed black line 'S_1'), we can model our delayed emission. We note that including this distribution does not significantly alter the full (TT) population (Figure S12). All the control thin films measured show similar dynamics.

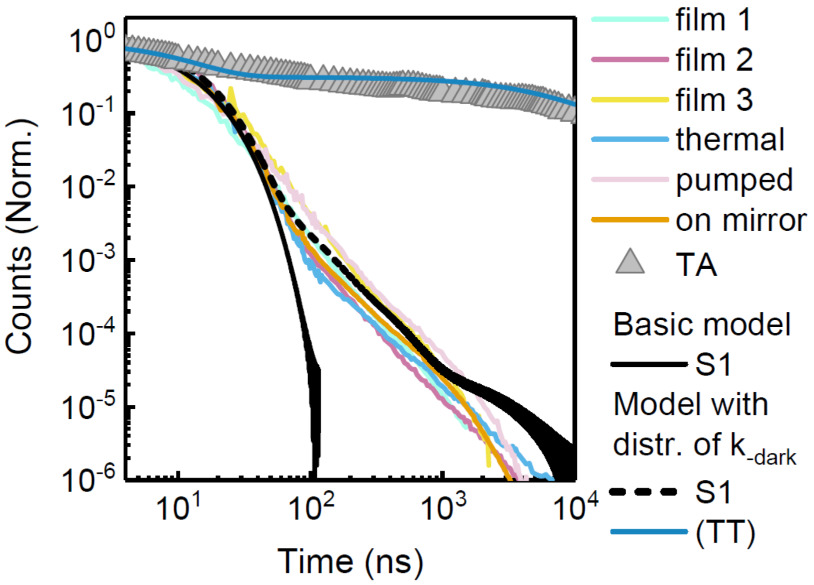

Figure S13. Model in Figure S12 applied to our data, which extends over many more orders of magnitude compared with the published PL data[8]. The delayed emission has been measured on numerous films, including those which are thermally annealed ('thermal'), have been pumped down under ultrahigh vacuum ('pumped') and have been deposited on a mirror ('on mirror'). With the basic model above we reproduce the exponential part of the S_1 delayed emission, but not the tail (black solid line). To reproduce the tail we used a distribution of k_{-dark} rates from $(30ns)^{-1}$ to smaller values. This is reasonable as TT_{dark} is made up of mixed singlet and quintet states and pure quintet states. The same process that can generate the pure quintet states can also produce pure singlet ^1TT, i.e. TT_{bright}, which should contribute to delayed S_1 emission. The distribution of rates could be due to the fluctuating nature of this population which moves from weak to strong coupling as the molecules vibrate relative to each other at room temperature. It could also be due to other effects such as the disordered nature of the sample.

Fitting to these complementary measurements over many orders of magnitude in time strongly constrains the few free parameters of our kinetic model. One of the central results of the model is that beyond ~70 ns the bulk of the excited-state population resides in the TT_{dark} state, and this state is dominant until the ~10 µs timescale. To describe our observations in the strong coupling regime, we assume that the essential photophysics of TIPS-tetracene are unperturbed in the exciton reservoir and use the same rate constants as for the film. Instead, we introduce the new emissive polariton state LPB. The low Q-factors of our cavities (30-60) give an extremely short intrinsic polariton lifetime, meaning that all dynamics will be governed by the exciton reservoir and transfer from it to the LPB, parameterised here as k_{pol_X} from each state. We stress that this phenomenological rate incorporates all processes that might contribute to the population transfer, including intra-reservoir effects such as exciton migration. For simplicity, we also assume in our model that the intrinsic decay of TIPS-tetracene results in quantitative population of the LPB and radiative emission.

From the fits in Figure S14, we find that the additional depletion of S_1 and TT_{bright} by the polariton (k_{pol_s} and k_{pol_b}) must be smaller than ~(100ns)$^{-1}$ in order for the pre-100ns decay not to be changed from the thin film reference. A faster (larger) rate would result in a significant shortening of the LPB emission lifetime compared with the bare film S_1 emission, which we do not observe. To fit the correct relative intensity of the microcavity-enhanced portion of the delayed emission we used k_{pol_d} = 0.005 k_{pol_s}. Using the same model but varying the ratio of k_{pol_s}/k_{pol_d}, we were able to fit microcavity emission with different detunings (Figure S14b).

This good fit of the model to our data and that of the literature suggests that the LPB emission beyond 100ns originates primarily from TT_{dark}, the equilibrium population of weakly exchange coupled singlet/quintet states and strongly exchange coupled pure quintet states. These states do not significantly contribute to emission in the pure film but are able to populate the LPB. We suggest the reason for this is the enhanced photonic mixing within TT_{dark}. Our model does not reproduce the tail of the LPB emission. At this point the triplet pairs are uncorrelated (but possibly still spatially bound through some polaronic effect[8]). The fraction of possible triplet pairs that could undergo enhanced photonic mixing (i.e. those which contain basis states $|xx\rangle$, $|yy\rangle$ or $|zz\rangle$ and hence some singlet character) has been reduced from 1 to 1/3.

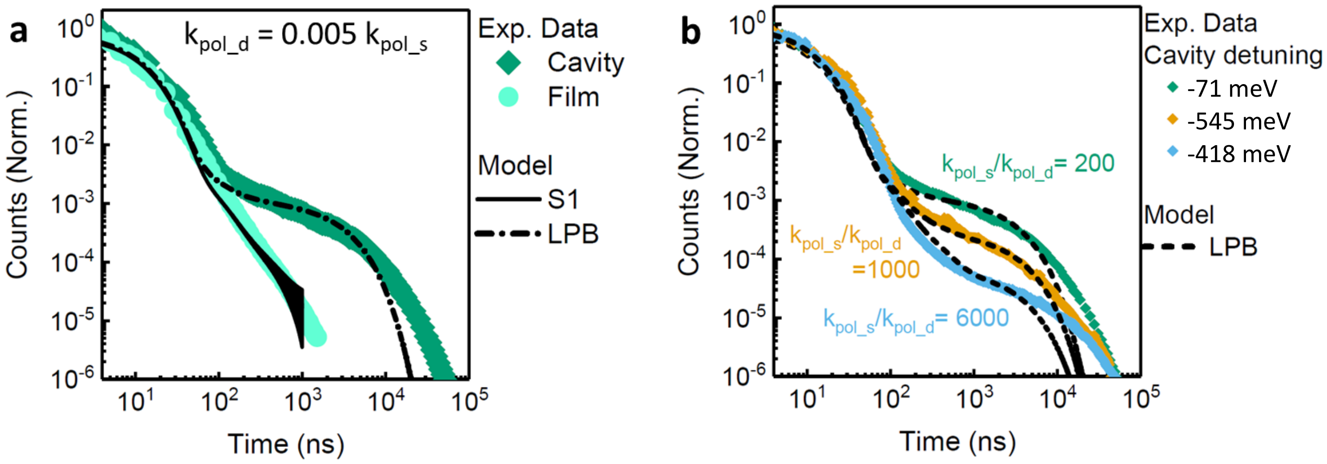

Figure S14. a Comparison between film and microcavity emission dynamics with the basic model in Figure S12a (rates in Table S2) including a distribution of k_{-dark} rates. The microcavity emission data is assumed to follow the LPB population. k_{pol_s} was set to (100ns)$^{-1}$ and k_{pol_d} was fit to (20μs)$^{-1}$ **b** Data from differently detuned cavities showing that the model reproduces the detuning by changing the relative rate of LPB population from S_1 and TT_{dark} (k_{pol_s} and k_{pos_d} respectively). The reason why detuning affects the relative rate of LPB population from TT_{dark} is not yet clear. The model does not match the very long-lived tail of emission (>10μs). At such long times, it is likely that either T_1+T_1 or free triplets populate the LPB via triplet-triplet annihilation. This has not been considered here.

We note that our model suggests an additional potential pathway of polariton population from TT_{dark}: indirectly, through an enhancement of k_{-dark} and consequent (polariton-enhanced) reformation of S_1. This would be an extremely surprising result, as it would suggest

that the intrinsic spin physics of uncoupled TIPS-tetracene states in the exciton reservoir are changed by light-matter coupling, but it must be considered. To probe this possibility, we performed the 'basic' model (without any distributions of rates) on the 'direct' and 'indirect' channels. For this we assumed no distribution of k_{-dark} (and therefore a poor fit to the film S_1 emission tail). In Figure S15a we show results for direct population of the LPB from TT_{dark} with, as above, k_{pol_d} = (20µs)$^{-1}$. In Figure S15b we show the best fit results for indirect population via TT_{bright} (assuming a microcavity-induced enhancement of k_{-dark} from 0 to (1000ns)$^{-1}$). Either mechanism would be interesting, but the better fit in Figures S15a and S14 compared with Figure S15b suggests direct population of LPB from TT_{dark} is the most likely mechanism.

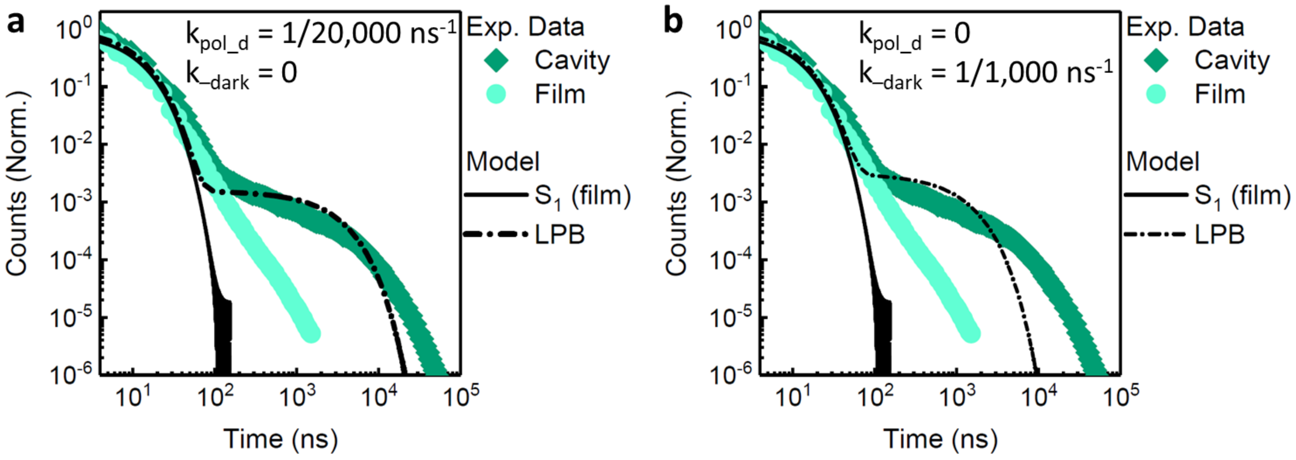

Figure S15. a Best fit to LPB emission using the model described above with k_{pol_s} = k_{pol_b} = 1/100ns^{-1} with all other parameters as for the model in Figure S12 (Table S2). **b** same model as in **a** but now with no direct population from TT_{dark} (k_{pol_d} = 0) and instead a fit for k_{-dark}=1/1000 ns^{-1}.

6. Kinetic enhancement

Though the level of emission in our experiments is low in the time domain, the total contribution from these long-lived states is significant. To illustrate this point, in Figure S16 we integrate the emission decay kinetics from the main text, normalising both curves for each material to the 'film' value for ease of comparison. Based on this simple metric, the degree of enhancement due to strong coupling (shaded) is 21% for DPPT, 64% for TIPS-tetracene and 133% for DPA. If we extend the integration in TIPS-tetracene and DPA cavities to longer delays, beyond the measurable lifetime of their reference films, the enhancement rises to 72% and 152%, respectively.

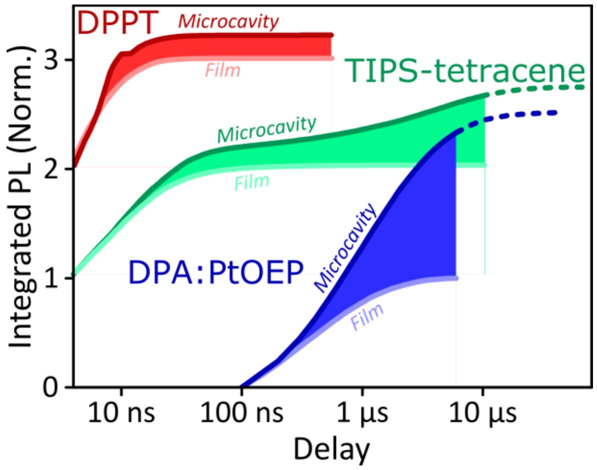

Figure S16. Time-integrated emission. Integral of emission kinetics in main-text Figures 2-4. For each material, both curves are normalised to the final value of the film integral, as a proxy for total film emission. Shaded region corresponds to the enhancement achieved through strong coupling. Dashed lines show continuation of microcavity emission beyond the range when film emission falls into noise.

7. References

1 S. A. Odom, S. R. Parkin and J. E. Anthony, *Org. Lett.*, 2003, **5**, 4245–4248.

2 C. Schweitzer and R. Schmidt, *Chem. Rev.*, 2003, **103**, 1685–757.

3 R. T. Grant, R. Jayaprakash, D. M. Coles, A. Musser, S. De Liberato, I. D. W. Samuel, G. A. Turnbull, J. Clark and D. G. Lidzey, *Opt. Express*, 2018, **26**, 3320.

4 R. Karpicz, S. Puzinas, V. Gulbinas, A. Vakhnin, A. Kadashchuk and B. P. Rand, *Chem. Phys.*, 2014, **429**, 57–62.

5 H. Goudarzi and P. E. Keivanidis, *J. Phys. Chem. C*, 2014, **118**, 14256–14265.

6 R. T. Grant, P. Michetti, A. J. Musser, P. Gregoire, T. Virgili, E. Vella, M. Cavazzini, K. Georgiou, F. Galeotti, C. Clark, J. Clark, C. Silva and D. G. Lidzey, *Adv. Opt. Mater.*, 2016, **4**, 1615–1623.

7 H. L. Stern, A. Cheminal, S. R. Yost, K. Broch, S. L. Bayliss, K. Chen, M. Tabachnyk, K. Thorley, N. Greenham, J. M. Hodgkiss, J. Anthony, M. Head-Gordon, A. J. Musser, A. Rao and R. H. Friend, *Nat. Chem.*, 2017, **9**, 1205–1212.

8 H. L. Stern, A. Cheminal, S. R. Yost, K. Broch, S. L. Bayliss, K. Chen, M. Tabachnyk, K. Thorley, N. Greenham, J. M. Hodgkiss, J. Anthony, M. Head-Gordon, A. J. Musser, A. Rao and R. H. Friend, *Nat. Chem.*, 2017, **9**, 1205–1212.

9 D. Ballarini, M. De Giorgi, S. Gambino, G. Lerario, M. Mazzeo, A. Genco, G. Accorsi, C. Giansante, S. Colella, S. D'Agostino, P. Cazzato, D. Sanvitto and G. Gigli, *Adv. Opt. Mater.*, 2014, **2**, 1076–1081.

10 D. M. Coles, P. Michetti, C. Clark, W. C. Tsoi, A. M. Adawi, J. Kim and D. G. Lidzey, *Adv. Funct. Mater.*, 2011, **21**, 3691–3696.

11 F. Herrera and F. C. Spano, *Phys. Rev. Lett.*, 2017, **118**, 223601.

12 F. Herrera and F. C. Spano, *Phys. Rev. A*, 2017, **95**, 053867.

13 L. R. Weiss, S. L. Bayliss, F. Kraffert, K. J. Thorley, J. E. Anthony, R. Bittl, R. H. Friend, A. Rao, N. C. Greenham and J. Behrends, *Nat. Phys.*, 2017, **13**, 176–181.

14 J. Aragó and A. Troisi, *Phys. Rev. Lett.*, 2015, **114**, 026402.

15 N. Nijegorodov, V. Ramachandran and D. P. Winkoun, *Spectrochim. Acta Part A Mol. Biomol. Spectrosc.*, 1997, **53**, 1813–1824.

16 J. J. Burdett, G. B. Piland and C. J. Bardeen, *Chem. Phys. Lett.*, 2013, **585**, 1–10.

17 H. L. Stern, A. J. Musser, S. Gelinas, P. Parkinson, L. M. Herz, M. J. Bruzek, J. Anthony, R. H. Friend and B. J. Walker, *Proc. Natl. Acad. Sci.*, 2015, **112**, 7656–7661.

18 C. K. Yong, A. J. Musser, S. L. Bayliss, S. Lukman, H. Tamura, O. Bubnova, R. K. Hallani, A. Meneau, R. Resel, M. Maruyama, S. Hotta, L. M. Herz, D. Beljonne, J. E. Anthony, J. Clark and H. Sirringhaus, *Nat. Commun.*, 2017, **8**, 15953.
